# Structure–Property Relationship of Defect-Trapped Pt Single-Site Electrocatalysts for the Hydrogen Evolution Reaction

**DOI:** 10.1021/acscatal.3c01513

**Published:** 2023-07-05

**Authors:** Peng Tang, Po-Yuan Huang, Jack E. N. Swallow, Chenbo Wang, Diego Gianolio, Hua Guo, Jamie H. Warner, Robert S. Weatherup, Mauro Pasta

**Affiliations:** † Department of Materials, University of Oxford, Parks Road, Oxford OX1 3PH, United Kingdom; ‡ Oxford Suzhou Centre for Advanced Research, 388 Ruoshui Road, Suzhou 215123, Jiangsu Province, P. R. China; ¶ Diamond Light Source Ltd., Harwell Science and Innovation Campus, Chilton, Didcot, OX11 0DE, U.K.; § Materials Graduate Program, Texas Materials Institute, The University of Texas at Austin, 204 East Dean Keeton Street, Austin, Texas, 78712, United States; ∥ Walker Department of Mechanical Engineering, The University of Texas at Austin, 204 East Dean Keeton Street, Austin, Texas, 78712, United States

**Keywords:** platinum single-site catalysts, in situ characterization, Pt-SSC formation mechanism, hydrogen evolution reaction, DFT calculations

## Abstract

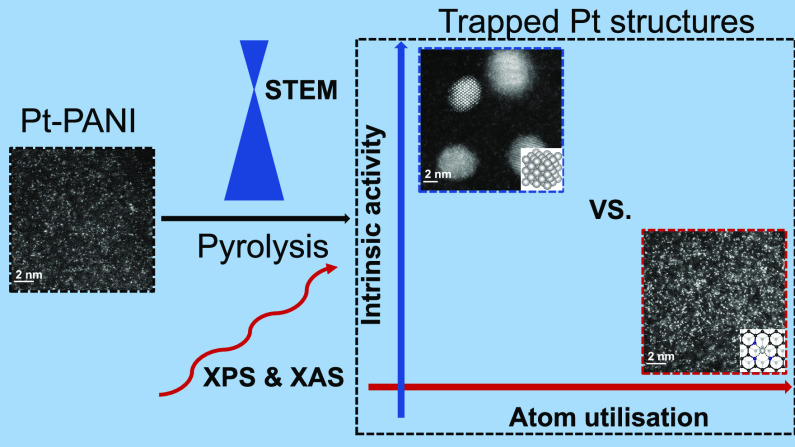

Single-site catalysts (SSCs) have attracted significant research interest due to their high metal atom utilization. Platinum single sites trapped in the defects of carbon substrates (trapped Pt-SSCs) have been proposed as efficient and stable electrocatalysts for the hydrogen evolution reaction (HER). However, the correlation between Pt bonding environment, its evolution during operation, and catalytic activity is still unclear. Here, a trapped Pt-SSC is synthesized by pyrolysis of H_2_PtCl_6_ chemisorbed on a polyaniline substrate. In situ heated scanning transmission electron microscopy and temperature-dependent X-ray photoelectron spectroscopy clarify the thermally induced structural evolution of Pt during pyrolysis. The results show that the nitrogen in polyaniline coordinates with Pt ions and atomically disperses them before pyrolysis and traps Pt sites at pyridinic N defects generated during the substrate graphitization. Operando X-ray absorption spectroscopy confirms that the trapped Pt-SSC is stable at the HER working potentials but with inferior electrocatalytic activity compared with metallic Pt nanoparticles. First principle calculations suggest that the inferior activity of trapped Pt-SSCs is due to their unfavorable hydrogen chemisorption energy relative to metallic Pt(111) surfaces. These results further the understanding of the structure–property relationship in trapped Pt-SSCs and motivate a detailed techno-economic analysis to evaluate their commercial applicability.

## Introduction

Proton exchange membrane (PEM) water electrolysis is one of the most promising technologies to generate green hydrogen from renewable energy sources.^1^ However, it relies on rare and expensive noble metal electrocatalysts, which undermines its economic viability.^2,3^ Single-site catalysts (SSCs), a new frontier in heterogeneous catalysis where isolated metal sites are supported on a substrate,^4−9^ have been proposed to alleviate this problem by maximizing noble metal utilization without compromising activity.^10,11^

Metallic Pt nanoparticles on a carbon support are the state-of-the-art hydrogen evolution reaction (HER) electrocatalyst in PEM electrolyzers.^12,13^ However, the current required mass loading of Pt nanoparticles may constrain large-scale (terawatt) deployment of PEM water electrolyzers.^3^ Highly dispersed Pt-SSCs have the potential to reduce the mass loading of Pt by maximizing atom utilization.^14,15^ Pt-SSCs trapped in defective carbon substrates have been reported to display higher mass activities for the HER compared to Pt nanoparticles as they fully expose the metal sites.^16−19^ However, their intrinsic catalytic activity relative to metallic nanoparticles is still a topic of debate in the field due to a lack of a detailed investigation into their structure–property relationship.^12,20,21^ Specifically, several reports have shown that Pt-SSCs on carbon substrates have higher mass activity than commercial Pt nanoparticles.^17,18,22^ On the contrary, other studies^20,23^ have suggested that Pt nanoparticles may be more active than oxidized Pt-SSCs for the HER. Thus, it is important to conduct a deep and fair evaluation of both Pt-SSCs and Pt nanoparticles for HER.

This fundamental interrogation requires a well-defined trapped Pt-SSCs structure that can then be used to investigate the bonding environment of Pt, its evolution under working conditions, and correlate it to catalytic activity and stability.^23−25^ One-pot pyrolysis, whereby a mixture of metal precursors and a carbon matrix are pyrolyzed at high temperatures (700–1000 °C), is commonly used to synthesize trapped SSCs.^26−30^ However, due to the complexity of the precursor mixture and the dynamic structural evolution of single sites induced by heating,^31,32^ a trial and error approach is commonly used to refine the synthesis conditions. A fundamental mechanistic understanding of the formation mechanism of Pt-SSCs is essential for the development of novel and reproducible synthesis methodologies.

Here we synthesize a Pt-SSC trapped in an N-doped graphene substrate by pyrolyzing H_2_PtCl_6_ chemisorbed on polyaniline (PANI).^33^ The formation mechanism of the trapped Pt-SSC is investigated by in situ heated scanning transmission electron microscopy (STEM) and temperature-dependent X-ray photoelectron spectroscopy (XPS) to probe the physical and chemical structures of Pt sites at various temperatures (25–800 °C). We show that N in PANI can coordinate with Pt-ions in the complex and trap Pt in the defects during substrate graphitization. After defining the bonding environment of the Pt-SSC with extensive in situ and ex situ characterization techniques, we investigate its evolution under working conditions using operando X-ray absorption spectroscopy (XAS). We show that the Pt-SSC structure is stable and that Pt remains in its (+2) oxidation state at the HER working potential. Finally, we compare the catalytic activity of our model Pt-SSC to that of Pt nanoparticles, with the model Pt-SSC demonstrating inferior catalytic properties. First principle calculations suggest that the inferior activity of the trapped Pt-SSC is due to its unfavorable hydrogen chemisorption energy relative to metallic Pt(111) surfaces.

## Results and Discussion

### Pt Single-Sites on Polyaniline

**Figure 1:**
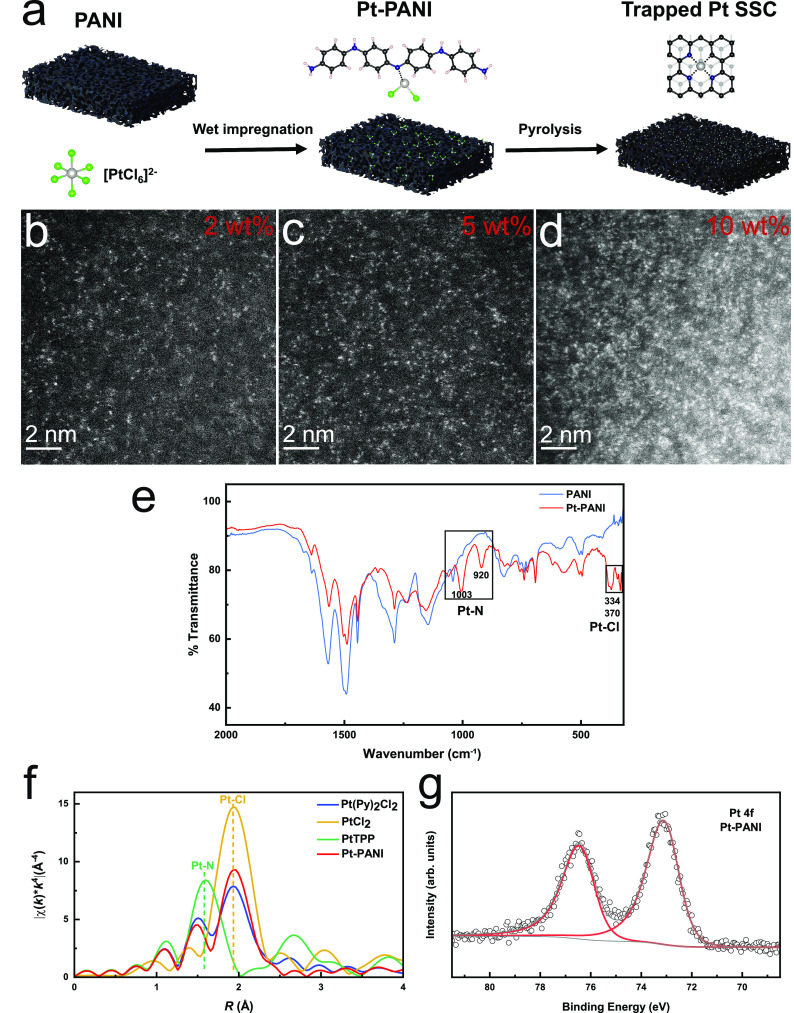
(a) Schematic of the synthesis process of Pt-PANI and trapped Pt-SSC. High-resolution ADF-STEM images of 2 wt% (b), 5 wt% (c) and 10 wt% (d) Pt-PANI shows Pt individually anchored on the PANI substrate. (e) FT-IR spectra of PANI and 10 wt% Pt single sites on PANI. (f) FT-EXAFS spectra of Pt(Py)_2_Cl_2_ (blue), PtCl_2_ (yellow), meso-tetraphenylporphine-Pt(II) (PtTPP) (green), and Pt-PANI (red). (g) XPS of Pt 4f for Pt-PANI.

A Pt-polyaniline (PANI) composite with well-dispersed Pt sites (Figure 1a) was synthesized via a wet impregnation method from H_2_PtCl_6_ and PANI (details in the experimental section). PANI is a commonly used substrate for the synthesis of SSCs because of its ability to coordinate with metal-ion complexes^34,35^ and hinder their aggregation.^36−38^ High-resolution annular dark-field (ADF) STEM images collected after the impregnation process (Figure 1b–d and Supplementary Figure S1) display Pt sites atomically dispersed on the polyaniline substrate with loading up to 10 wt%. The density of isolated Pt sites in 2 wt%, 5 wt% and 10 wt% samples are ∼3, ∼7 and ∼14 nm^–2^, respectively (as determined by calculating the number of bright dots in the STEM images which are representative of each sample).

The interaction between  and PANI was investigated by Fourier-transform infrared spectroscopy (FT-IR) and XAS. As shown in the FT-IR spectra of the 10 wt% Pt-PANI sample (Figure 1e), in addition to the characteristic peaks of PANI, new peaks corresponding to Pt-Cl and Pt-N vibrations are present at 334/370 cm^1^ and 920/1003 cm^1^, respectively.^39^ These new vibrations indicate that Pt-ions are anchored on the PANI substrate by interactions with N; the remaining Cl ligands are from the precursor. To provide further information on the bonding environment of Pt, EXAFS was performed at the Pt L_3_-edge and compared with reference Pt structures. As shown in Figure 1f, both the Fourier-transformed (FT)-EXAFS spectra of the Pt-PANI (red) and Pt(Py)_2_Cl_2_ (blue) feature two peaks at 1.93 and 1.50 Å, which are in agreement with the positions of the Pt-Cl bond in PtCl_2_ (yellow) at 1.93 Å and Pt-N bond in meso-tetraphenylporphine-Pt(II) (PtTPP, green) at 1.56 Å, respectively. The EXAFS data provide further evidence that Pt single sites bond with N in the PANI substrate and remaining Cl ligands.

The oxidation state of Pt in the Pt-PANI sample is further clarified by XPS and XANES. A Pt 4f_7/2_ peak with a binding energy of 72.9 eV, corresponding to an oxidation state of + 2,^33^ is present in the Pt core level XPS spectrum of Pt-PANI (Figure 1g). The XANES of Pt-L_3_ in Pt-PANI and Pt(Py)_2_Cl_2_ have similar edge-line position and white-line intensity (Figure S4), which also indicates the oxidation state of Pt in the Pt-PANI sample is + 2. Pt is partially reduced from a + 4 to a + 2 oxidation state as it transitions from the H_2_PtCl_6_ precursor to become anchored on the PANI substrate. The presence of metal-bonded Cl peaks at 198.3 eV from Pt-Cl in the Cl 2p spectra can also prove the successful anchoring of Pt sites (Supplementary Figure S5). Combining the STEM, FT-IR, EXAFS and XPS data, we confirm that Pt-ions are well dispersed throughout the sample due to their coordination to the aniline N in PANI.

### Trapped Pt-SSC on N-Doped Graphene

**Figure 2:**
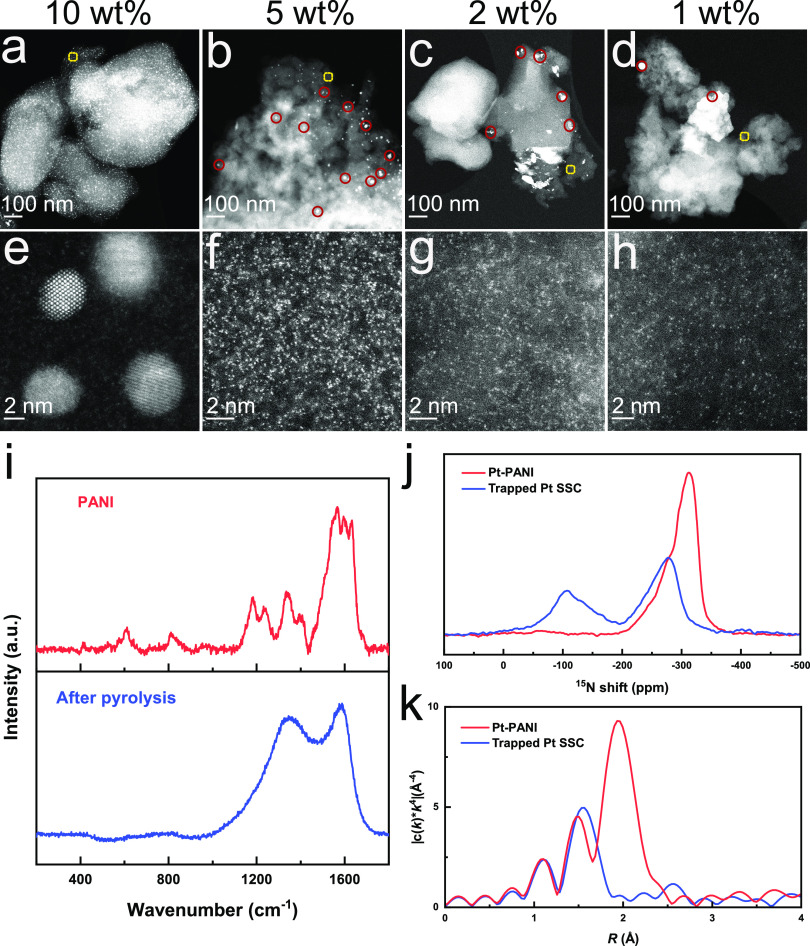
Low- (a–d) and high-resolution (e–h) STEM images of Pt-PANI with various loadings after pyrolysis in N_2_ at 800 °C for 1.5 h, which are representative of each sample; 10 wt% (a, e), 5 wt% (b, f), 2 wt% (c, g) and 1 wt% (d, h). The red circles highlight the formed nanoparticles, and the yellow rectangles outline the areas where high-resolution STEM images were recorded (e–h). (i) Raman spectra of the PANI substrate before and after pyrolysis. CPMAS NMR spectra of ^15^N (j) and FT-EXAFS (k) of 1 wt% Pt-PANI before and after pyrolysis in N_2_ at 800 °C for 1.5 h.

Pt-PANI samples with various Pt loadings (10 wt%, 5 wt%, 2 wt% and 1 wt%) were pyrolyzed at 800 °C under N_2_ for 1.5 h and charactrized by ex situ STEM (Figure 2a–h). Pt nanoparticles can be identified in the pyrolyzed Pt-PANI samples with Pt loadings of 10 wt% (Figure 2a,e), 5 wt% (Figure 2b,f), and 2 wt% (Figure 2c,g), mainly at the edge of the substrates. More specifically, the 10 wt% Pt-PANI sample is covered with 3–5 nm nanoparticles remain after pyrolysis (a), while there are ∼150 and ∼42 nanoparticles by counts in the b (5 wt%) and c (2 wt%) samples, respectively. At the lowest Pt loading of 1 wt%, almost all the Pt forms a trapped single-site (Figure 2d,h). Therefore, we selected the 1 wt% Pt-PANI sample as a model for further investigation (see discussion in Supplementary Figure S2).

Raman spectroscopy and cross-polarization magic-angle spinning nuclear magnetic resonance spectroscopy (CPMAS NMR) were used to confirm the structure of the PANI after pyrolysis. The ex situ Raman spectra of PANI before and after pyrolysis (Figure 2i) show that all the characteristic PANI peaks vanish after pyrolysis, while two new peaks at 1360 and 1580 cm^–1^ appear which are from the D and G bands of graphene. This indicates the formation of a defective graphene substrate.^40^ Furthermore, the new peaks formed after pyrolysis in the ^15^N CPMAS NMR spectra (Figure 2j), ranging from −50 to −200 ppm, demonstrate the formation of pyridinic N and graphitic N.^41^ The Raman and NMR data together suggest that the PANI substrate becomes N-doped graphene after pyrolysis.

Figure 2k shows the Pt L_3_-edge EXAFS spectra of the Pt-SSC before and after pyrolysis. After pyrolysis, the Pt-Cl peak at 1.93 Å disappears, leaving the peak at 1.58 Å (Pt-N) as the main peak. The absence of a significant Pt-Pt peak at 2.73 Å means that the Pt sites remain isolated and bonded to N as trapped Pt-SSCs.

### Formation Mechanism of Trapped Pt-SSC

**Figure 3:**
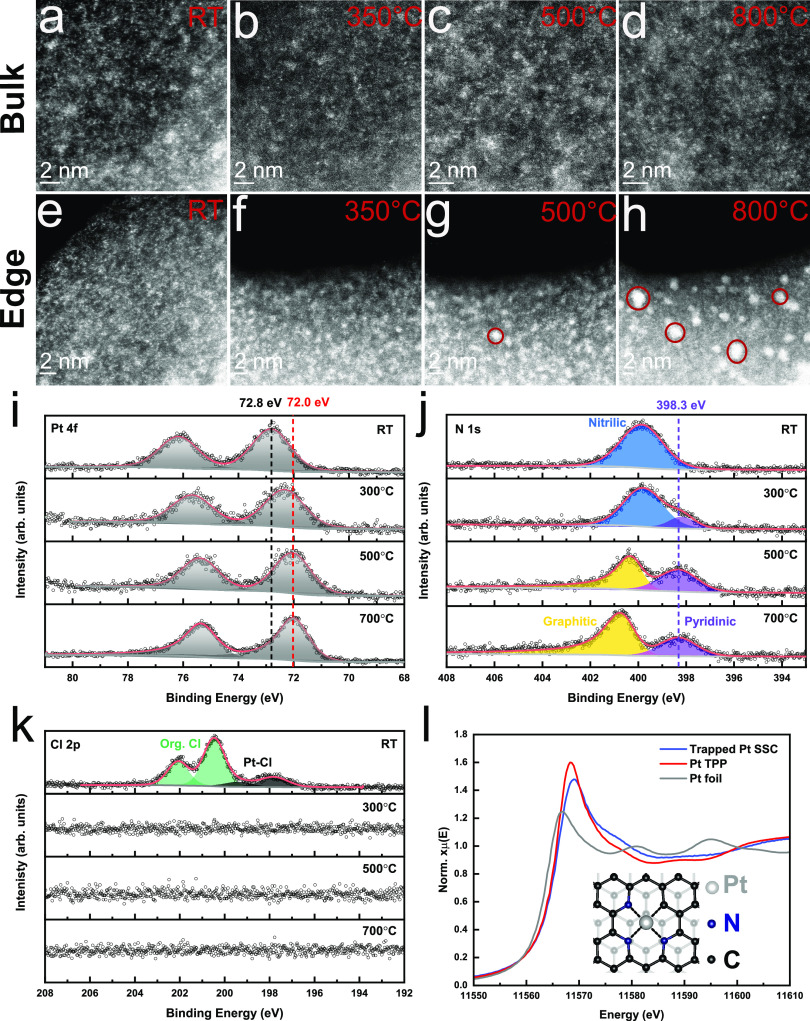
In situ heating STEM images showing the spacial evolution of Pt sites at the bulk (a–d) and edge (e–h) in a sample with 10 wt% Pt on PANI with increasing temperatures (RT, 350 °C, 500 °C, and 800 °C) under vacuum. (i–k) Temperature-dependent XPS data of 1 wt% Pt-PANI under vacuum to show how the bonding environment of Pt evolves during pyrolysis. Pt 4f spectra (i), N 1s spectra (j), and Cl 2p spectra (k). (l) XANES of the trapped Pt-SSC synthesized by pyrolysis, compared with the Pt TPP complex and Pt foil. The inset is a schematic of the trapped Pt-SSC structure; black for carbon, blue for N, and gray for Pt.

In situ heating STEM was used to track the spacial evolution of the Pt sites in the 10 wt% Pt-PANI sample with increasing temperature to form trapped Pt-SSC. The majority of the Pt sites in the bulk remain isolated even when the temperature reaches 800 °C (Figure 3a–d and Supplementary Figure S11). On the contrary, some of the Pt sites along the edge (Figure 3e–h and Supplementary Figures S12 and S13) start to aggregate at 500 °C and further transform into nanoparticles at 800 °C (Figure 2a,e). The formation of Pt nanoparticles confirms that Pt sites tend to aggregate at high temperatures, especially with relatively high Pt loadings.

Temperature-dependent XPS was then performed to track the changes in the bonding environment of Pt in a 1 wt% Pt-PANI sample (10 wt% in the Supplementary Figure S14) core level for Pt 4f, N 1s, Cl 2p, C 1s, and O 1s were recorded. As Figure 3i shows, the Pt 4f peaks shift to lower binding energies with increasing temperature; the Pt 4f_7/2_ shifts from 72.8 eV (Pt(+2)) at room temperature (RT) to 72.0 eV at 700 °C. This suggests a change in Pt’s chemical environment as the pyrolysis temperature increases, possibly toward more metallic Pt. Further, the Pt 4f peaks exhibit a more asymmetric peak shape at 700 °C, which is indicative of this more metallic Pt bonding environment. More importantly, as the temperature increases to 700 °C, the N 1s peak gradually splits into two peaks: a graphitic N peak at 400.9 eV and a pyridinic N peak at 398.3 eV (Figure 3j).^33,42^ This significant change indicates the graphitization of the PANI substrate at high temperatures, which is also supported by the shifts in the C-N peak at C 1s (Supplementary Figure S15). In comparison, the Cl spectra show that peaks from Cl bonded to Pt sites at 198.3 eV and organic Cl peaks at 200 eV from PANI at RT disappear by the time the samples reach 300 °C (Figure 3k). Oxygen peaks also disappear at this temperature (Supplementary Figure S15).

Overall, as can be seen in survey scans (Supplementary Figure S16), although the intensity of the N peaks has dropped, the disappearance of the Cl and O peaks indicates that only the remaining N can continuously bond with the Pt to trap it at high temperatures. Based on the different electronic density of N sites, it is more likely that pyridinic N, rather than graphitic N, donates electrons to bond with Pt sites.^43^ By comparing the XANES data of the trapped Pt-SSC with PtTPP and Pt foil (Figure 3l), we can confirm that the Pt sites in trapped Pt-SSC have a similar oxidation state and bonding environment as Pt(II) in the PtTPP complex, which is consistent with our XPS results (Figure 3i). Therefore, we fit the EXAFS data in Figure 2k with calculated DFT models (see discussion in Supplementary Figure S7 and Table S2), we propose the following atomic structure for trapped Pt-SSC: one Pt site bonds with 3 N and 1 C on the N-doped graphene (Pt-N_3_-C structure of the inset in Figure 3l).

In summary, in the Pt-PANI samples, Pt sites are atomically bonded with N and Cl, yielding a formal oxidation state of +2. During the pyrolysis process, they are then gradually donated electrons by the pyridinic N sites and finally become trapped in the pyridinic N defects formed during the substrate graphitization. Pt-sites in Pt-PANI samples with higher Pt loading are prone to reduction to their metallic state and aggregation into nanoparticles, especially at edge areas (Figure 3h), when the surrounding N sites are evaporated at high temperatures.

### HER Activity of Trapped Pt-SSC

After clarifying the structure of the trapped Pt-SSC (1 wt% Pt-PANI after pyrolysis), its catalytic activity for the HER was evaluated using a customized three-electrode setup and compared with other trapped Pt-SSC samples (5 and 10 wt% Pt-PANI after pyrolysis) and commercial 20 wt% Pt/C (1 μg_Pt_ cm^–2^). The three-electrode setup is specially designed to minimize the mass transfer limitations in the HER (Supplementary Figure S17 and Supplementary Figure S18), which is critical for an accurate evaluation of the HER catalytic activity. Their activities were analyzed using cyclic voltammetry (CV) at a scan rate of 10 mV s^–1^ in 0.5 M H_2_SO_4_ saturated with pure H_2_. Representative structures of the trapped Pt samples (1 wt%, 5 wt% and 10 wt% Pt-PANI after ex situ pyrolysis) and commercial 20 wt% Pt/C are shown as STEM images in Figure 2 and Supplementary Figure S24. As previously discussed, the trapped 1 wt% Pt sample (trapped Pt-SSC) is a single-site structure, the trapped 10 wt% Pt sample is composed of well-dispersed nanoparticles and single sites, and the 20 wt% Pt/C sample is covered with nanoparticles with an average size of 2–3 nm (Supplementary Figure S24).

**Figure 4:**
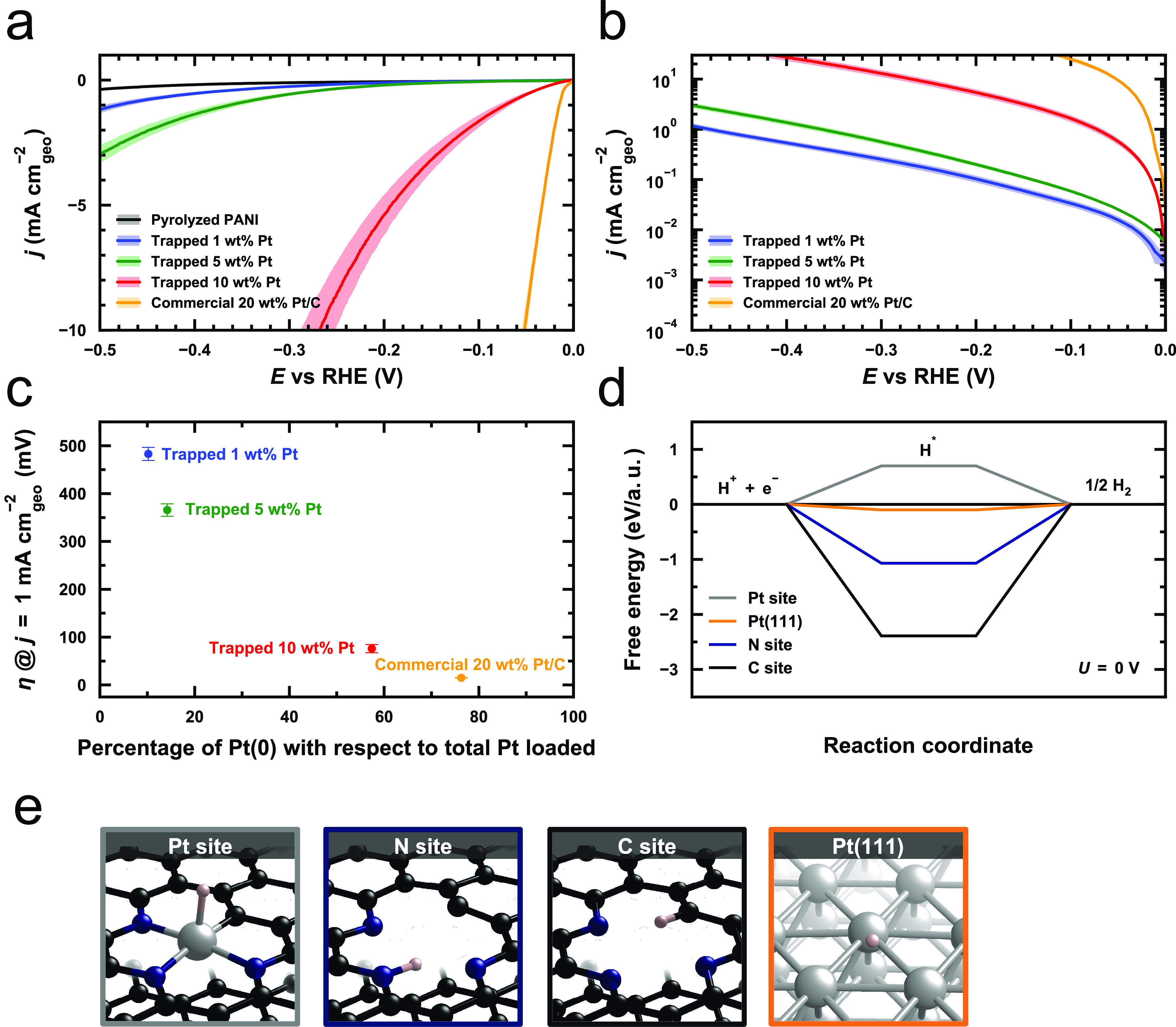
(a) HER polarization curves for trapped Pt-SSC samples synthesized by one-pot pyrolysis and commercial 20 wt% Pt/C in 0.5 M H_2_SO_4_ electrolyte, showing the overpotential needed to reach a specific geometric current density. Solid curves represent the averaged current response obtained from a minimum of three separate measurements, and the shaded regions show the range of standard errors relative to the mean values. (b) The areal current density on a logarithm-10 scale derived from the average CV curves for Pt samples in (a). (c) The correlation between the onset overpotentials (η at the current density of 1 mA cm^–2^) and the percentage of Pt(0) with respect to total Pt loaded in an ex situ pyrolyzed Pt samples. (d) Free energy diagram for the hydrogen evolution at the reaction potential U = 0 V. The data are calculated at 1 bar of H_2_ and pH = 0 at 298 K. (e) Relaxed structures of hydrogen adsorbed on Pt site, N site, C site, and the FCC site on Pt(111) surface. Pink for hydrogen, black for carbon, blue for N, and metallic for Pt.

From the CV curves shown in Figure 4a, the trapped 1 wt% Pt sample (trapped Pt-SSC, blue line) has a much higher onset overpotential than the other samples (the overpotential to reach a current density of 1 mA cm^–2^); the 1 wt% sample requires 483.0 ± 13.7 mV compared with only 15.0 ± 0.5 mV for the Pt/C (yellow). Electrochemical active surface area (ECSA) specific current density and Pt-mass specific current both show the same activity trend (Supplementary Figure S20). The HER activities of trapped Pt samples increase significantly with Pt loading (from blue 1 wt% to green 5 wt% to red 10 wt%). The trapped 10 wt% Pt sample, which contains vastly more nanoparticles, shows superior activity, needing only 76.0 ± 8.6 mV vs RHE to reach a current density of 1 mA cm^–2^, as seen in Figure 4a. This is 407 mV and 290 mV lower than the trapped 1 wt% and 5 wt% samples, respectively.

Furthermore, Figure 4b and the corresponding Tafel analysis (Supplementary Figure S21) show a comparison of the HER kinetics by calculating the base-10 logarithm of the geometric current density in Figure 4a. In Pt single site-dominated samples (trapped 1 wt% and 5 wt% Pt) are much slower than in Pt nanoparticle-dominated samples (trapped 10 wt% Pt and 20 wt% Pt/C). This indicates that the trapped Pt-SSCs may have a different reaction mechanism for the HER compared to that of the metallic Pt nanoparticles.^44^ Correlation between the oxidation state of Pt (Supplementary Figure S25) and the HER activity is demonstrated in Figure 4c. The more reduced Pt structure with a higher number of nanoparticles has a lower onset overpotential for the HER, which is consistent with metallic Pt being more active than the oxidized trapped Pt-SSCs trapped on the N-doped graphene.^20^ Combining STEM, XPS and HER data, we conclude that the model trapped Pt-SSCs for the HER show inferior HER activity than metallic nanoparticles. This is evidenced by comparing the HER performance of 1 wt% trapped Pt-SSC, an oxidized Pt single-site sample, with the commercial 20 wt% Pt/C, a metallic Pt-nanoparticle structure (further discussion in Supplementary Figure S22).

The origin of the low HER activity of the trapped Pt-SSCs can be probed using a first-principles approach (Figure 4d). We calculate the hydrogen chemisorption free energy using density-functional theory (DFT) at an applied external potential of U = 0 V and pH = 0 on the Pt, C, and N sites of the Pt-N_3_-C model shown in Figure 4e. The hydrogen chemisorption free energy is an indicator of the HER activity for Pt group metals, with values closest to 0 eV showing the highest HER activity.^45^ A 0.70 eV hydrogen chemisorption energy for the Pt site is observed (gray line and gray frame in Figure 4d and e). This value is further from the thermo-neutral value than that of the Pt(111) surface (−0.10 eV), implying that the occurrence of the HER may be less favorable on the single Pt site than on the metallic Pt(111) surface.^23,46^ Strong hydrogen adsorption on the N (−1.07 eV, the blue line in Figure 4d and e) and C site (−2.39 eV, black line in Figure 4d and e) can also be observed, suggesting that these two sites are unlikely to catalyze the HER. The poorer activity of the Pt-SSC can be attributed to the lower electron density available for protons at the Pt site relative to that in Pt metal or intermediate nanoclusters. These observations are in agreement with our experimentally measured activities.

### Structure of Trapped Pt-SSC at Working Potentials

**Figure 5:**
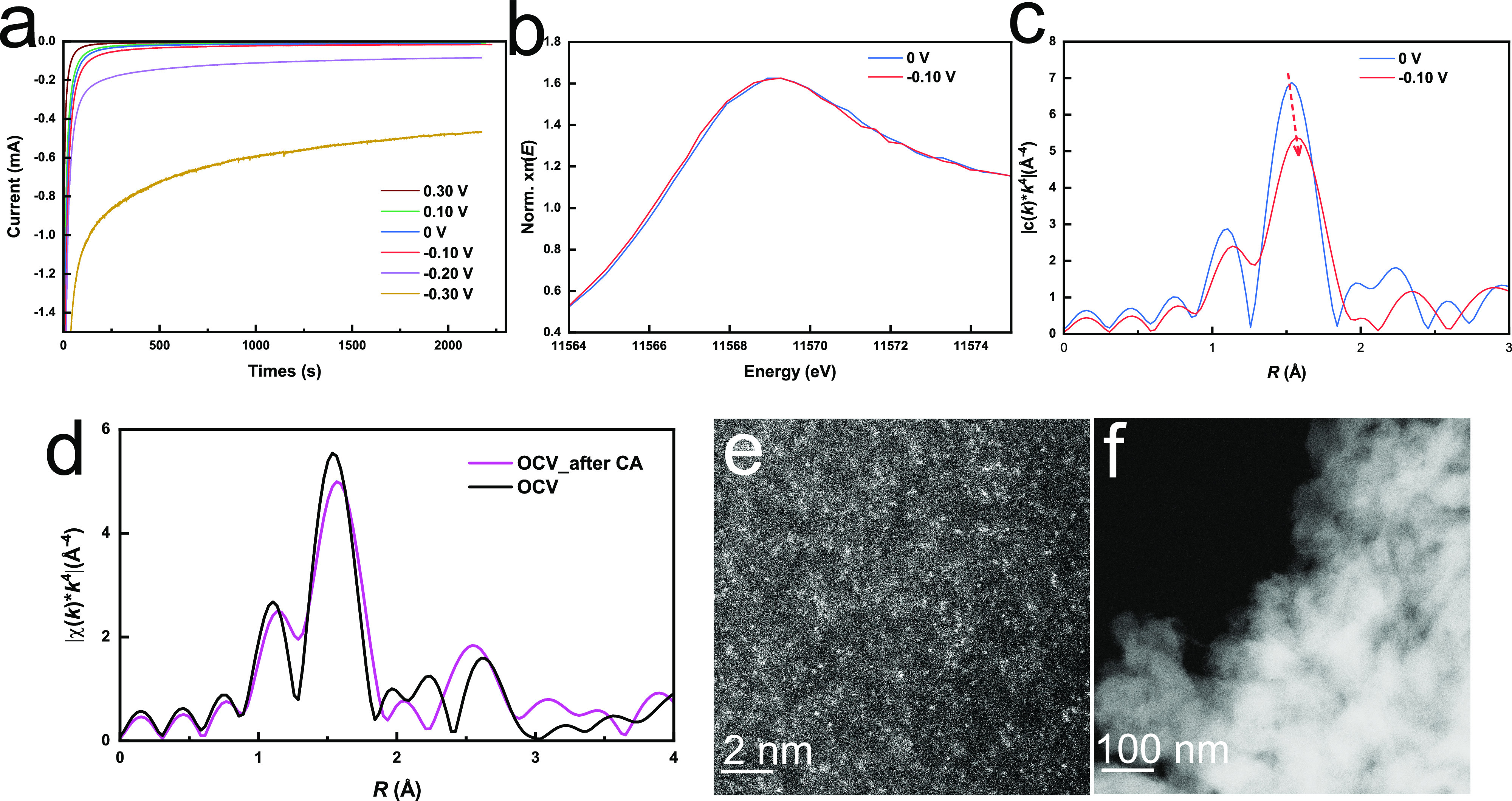
(a) Chronoamperometry curves of trapped Pt-SSCs at different applied potentials. (b) and (c) XANES and FT-EXAFS spectra of the Pt L_3_-edge for the trapped Pt-SSCs highlighted at 0 and −0.10 V. (d) FT-EXAFS spectra of the Pt-L_3_ edge for trapped Pt-SSCs at the OCV before and after CA tests. (e) and (f) Atomic STEM images of trapped Pt-SSC after 2 h of CA test at −0.2 V vs RHE with a scale bar of 2 and 100 nm, respectively, showing no Pt nanoparticle can be observed after the standard stability test.

Finally, operando XAS was employed to investigate the structure of the trapped Pt-SSCs under working potentials for the HER. Operando XAS spectra of the Pt L_3_-edge were recorded alongside the current when various potentials were applied to the sample (from 0.50 V (OCV) to −0.30 V vs RHE). The setup of the operando experiment is shown in Supplementary Figure S30. The chronoamperometry (CA) curves recorded at 7 potentials are shown in Figure 5a, revealing that hydrogen starts to evolve significantly below −0.1 V vs RHE. From the FT-EXAFS spectra of the Pt L_3_-edge at all potentials (Supplementary Figure S28), the peak at 1.50–1.60 Å, representing the Pt-N bond, is dominant throughout the experiment. No Pt-Pt peak is observed, but some distortion of the Pt-N peak with decreasing potential is apparent.

From OCV to 0 V vs RHE, there is no shift in the absorption edge and the white-line intensity of the XANES spectra (Supplementary Figure S28), indicating no significant change in the oxidation state of Pt above 0 V. A slight reduction of Pt is found from 0 to −0.1 V with the white line shifting negatively by 0.1 eV (Figure 5b) when the HER occurs. An apparent distortion in the Pt-N peak in the FT-EXAFS spectra can also be observed at −0.1 V in Figure 5c. Fitting the operando EXAFS data with the proposed Pt-N_3_-C structure (Supplementary Table S3) suggests that this corresponds with a shortening of the Pt-N bond by ∼ 0.01 Å. The slight reduction of Pt and the decrease in the Pt-N bond length indicate that the trapped Pt-SSCs have been reduced slightly to initiate the reaction.

At the working potentials for the HER (−0.1 V, −0.2 V, and −0.3 V vs RHE), the oxidation state of Pt remains stable, as shown by the XANES spectra in Supplementary Figure S28c, and lack of observed Pt-Pt peak at 2.70 Å (Supplementary Figure S28a). The data show that Pt remains stable in a single-site structure under HER working conditions. Even when the potential returns to the OCV after the CA tests, the Pt-N bonding is retained (Figure 5d and Supplementary Figure S29). The stability of the trapped Pt-SSCs is further proven by the ADF-STEM images of the used catalyst. As shown in Figure 5e, f, no Pt aggregates are recorded after performing the CA test for 2 h at −0.2 V vs RHE. Overall, the data show that the trapped Pt-SSCs retain a stable structure under an HER working potential, with no aggregation occurring during normal operation.

## Conclusions

In this work, model-trapped Pt-SSCs on N-doped graphene were prepared by pyrolyzing atomically dispersed Pt sites on a PANI substrate. In situ characterization experiments (STEM and XPS) were combined to clarify the formation mechanism of trapped Pt-SSCs. We found that N sites on the substrate could persistently bond with Pt sites during pyrolysis and trap Pt as isolated sites in pyridinic N defects after graphitization of the substrate. By combining in situ characterization data with complementary ex situ characterization results (XAS, Raman, and CPMAS NMR), the atomic structure of the trapped Pt-SSCs was identified: Pt(+2) single sites bond with three pyridinic Ns and one carbon on the N-doped graphene substrate. The local structure of Pt sites of trapped Pt-SSC is an analogue of Pt macrocyclic Pt(II) complexes.

Next, the intrinsic activity of the model trapped Pt-SSCs for the HER was clarified. HER polarization curves show that an additional 468 mV is required for the trapped 1 wt% Pt-SSC to reach the current density of 1 mA cm^–2^ compared with commercial 20 wt% Pt/C, a metallic Pt-nanoparticle structure. By correlating the activity and oxidation state of our Pt catalysts and commercial Pt nanoparticles, we confirmed that a metallic Pt structure is more active for the HER because of its lower hydrogen chemisorption energy. We believe this is due to Pt-SSCs having a lower electron density relative to metallic Pt nanoparticles, which is attributed to their oxidized state in this bonding environment. The operando XAS experiment shows a trapped Pt-SSC is indeed stable at the HER working potentials. While metallic Pt single-site structures that are stable under the HER conditions have not been well-demonstrated to date, our study suggests that a detailed techno-economic analysis is needed to evaluate if the benefits of the reduced metallic loading and long-term stability of trapped Pt single-sites compared to state-of-the-art Pt nanoparticles outweigh their lower catalytic activity and more complex synthetic procedure.

## Data Availability

The authors declare that all data supporting the findings of this study are included within the paper and its Supporting Information files. Source data are available from the corresponding author upon reasonable request.

## Supporting Information Available

Experimental method and materials; XANES and XPS spectra of Pt-PANI; additional temperature-dependent XPS spectra of 1 wt% Pt-PANI; size distribution of Pt single sites and Pt nanoparticles of trapped 1 wt% Pt-SSC; ex situ and operando EXAFS analysis of trapped 1 wt% Pt-SSC, including photograph of the operando XAS setup; discussions on the protocol of HER testing and the catalytic and DFT calculation results (PDF)
