# Supplementary material for: Structure–Property Relationship of Defect-Trapped Pt Single-Site Electrocatalysts for the Hydrogen Evolution Reaction
Source: ACS Catal. 2023 Jul 5;13(14):9558–66. doi: 10.1021/acscatal.3c01513 (PMC10367054; doi:10.1021/acscatal.3c01513)
Supplement: Supplementary file 1 — cs3c01513_si_001.pdf [file cs3c01513_si_001.pdf]

SUPPLEMENTARY INFORMATION

Structure-Property Relationship of Defect-Trapped Pt Single-Site Electrocatalysts for the Hydrogen Evolution Reaction

Peng Tang,[†,⊥] Po-Yuan Huang,[†,⊥] Jack E. N. Swallow,[†] Chenbo Wang,[‡] Diego Gianolio,[¶] Hua Guo,[†] Jamie H. Warner,[§,‖] Robert S. Weatherup,[†] and Mauro Pasta*,[†,‡]

†*Department of Materials, University of Oxford, Parks Road, Oxford OX1 3PH, United Kingdom*

‡*Oxford Suzhou Centre for Advanced Research, 388 Ruoshui Road, Suzhou 215123, Jiangsu Province, P. R. China*

¶*Diamond Light Source Ltd., Harwell Science and Innovation Campus, Chilton, Didcot, OX11 0DE, U.K.*

§*Materials Graduate Program, Texas Materials Institute, The University of Texas at Austin, 204 East Dean Keeton Street, Austin, Texas, 78712, United States*

‖*Walker Department of Mechanical Engineering, The University of Texas at Austin, 204 East Dean Keeton Street, Austin, Texas, 78712, United States*

⊥*P.T. and P-Y.H. contributed equally*

E-mail: mauro.pasta@materials.ox.ac.uk

Experimental Section

Chemicals

Chloroplatinic acid hexahydrate (37.5% Pt basis), aniline (99.5%), ammonium persulfate (APS) (98%) and Supelco Suprapur H_2SO_4 were purchased from Sigma-Aldrich. Ultra-pure water (18.2 MΩ cm^{-1}, Direct-Q Water Purification System) was used for the electrochemical tests. N5.5 grade hydrogen gas for puring of the electrolyte was purchased from BOC.

Catalysts synthesis

The Pt-PANI samples with varied Pt loading were synthesized by a wet impregnation method. Firstly, polyaniline was prepared via the oxidative polymerisation of aniline monomer with APS.[1] Then, the milled polyaniline was dispersed in ethanol solution and ultrasonicated for 30 mins; 10 mM H_2PtCl_6 dissolved in ethanol was transferred dropwise into the polyaniline solution under vigorous stirring for 4 h. Finally, the mixed solution was centrifuged at 10,000 rpm for 10 mins (repeated two or three times) to obtain the dry Pt-PANI powder after evaporating at 60 °C to remove the ethanol solvent. The trapped Pt SSC catalysts were prepared by pyrolysing Pt-PANI dry powder samples. Specifically, the Pt-PANI was placed in a tube furnace, and the temperature was increased from RT to 800 °C at a ramp rate of 15 min °C^{-1}. After reaching the setpoint temperature (800 °C), the Pt-PANI was annealed at 800 °C for 1.5 hrs to graphitize completely. The furnace was then left to cool naturally to obtain the final desired trapped catalysts.

Ex-situ STEM and in-situ heated STEM

Room-temperature annular dark-field scanning transmission electron microscopy was performed with a JEOL ARM200F at an accelerating voltage of 200 kV. Dwell times of 10-20 µs, a beam current of 35 pA, and a pixel size of 0.006 nm px^{-1} were used for imaging, with a convergence semi-angle of 25.5 mrad and collecting inner-outer angles 68 to 275 mrad. The

in-situ STEM experiments were performed at a lower accelerating voltage of 80 kV to minimise the beam damage to the system. An in-situ holder with a software-controlled heating chip developed by Dens solution company was used to deposit a 10 wt% Pt-PANI sample.

Ex-situ XPS and temperature-dependent XPS

X-ray photoemission spectroscopy was performed using a PHI VersaProbe III system generating monochromatic Al X-rays at 1486 eV. A photoelectron take-off angle of 45° was used, and all measurements were performed at an X-ray power of 25 W and a chamber pressure less than 4×10^{-9} mbar. Survey measurements were performed at a pass energy of 224 eV. In comparison, core levels were measured at a pass energy of 13 eV (26 eV), giving an energy resolution of 0.4 eV (0.45 eV) (determined from fitting a Fermi function convoluted with a Gaussian to the Fermi edge of a sputtered Au foil). The beam spot was circular with a nominal diameter of 100 µm. Samples were deposited directly onto gold foil for XPS measurements which negated the need for any charge compensation mechanisms.

For temperature-dependent XPS experiments, samples were transferred into a prep-chamber without breaking the vacuum to be heated (pressure in prep-chamber was 10^{-8} mbar). Subsequently, samples were moved back into the XPS system through a load lock (pressure of 10^{-6} mbar). In-situ heating was performed using a ceramic (pyrolytic graphite and boron nitride) heater from Thermic Edge, capable of reaching 1600 °C in UHV. A type K thermocouple was used to contact the Mo sample plate during heating to allow temperature measurements.

The binding energies were calibrated using the C 1s line at 284.8 eV in Fig. 1 or Au 4f peak as references in Fig. 3i, j, k and Fig. 4c. The XPS spectra were fit using Gaussian-Lorentzian and Voigt lineshapes (where asymmetry was pronounced), and quantified with the help of CasaXPS software.

Raman and FT-IR

The Raman spectra were collected with a Renishaw InVia microscope using a 532 nm laser. Multiple acquisitions were made with a 1% laser intensity for 1 s exposure time. The FT-IR spectra of the as-synthesized PANI, Pt-PANI, and Pt-PANI after pyrolysis were obtained using a Thermo Scientific Nicolet 6700 on an attenuated total reflection (ATR) stage in a nitrogen-filled glovebox.

Solid State Magic Angle Spinning NMR

All solid-state NMR experiments were performed on a 9.45 T Bruker Avance III HD NMR spectrometer, operating at ^{1}H and ^{15}N Larmor frequencies of 399.89 and 40.52 MHz respectively. A Bruker 4 mm double-resonance magic angle spinning (MAS) probe was used to facilitate a spinning frequency of 12 kHz. For the direct observation experiments, a 5.5 µs pulse and 100 kHz ^{1}H SPINAL-64 heteronuclear decoupling were utilised, whilst for the cross-polarisation experiments a 2.5 ms ramped (70-100 %) contact pulse was employed. A minimum of 2048 transients were recorded for each spectrum with a recycle delay of 3 seconds. The ^{15}N chemical shifts were referenced indirectly to neat liquid nitromethane (CH_3NO_2, 0 ppm) by using the secondary reference of powdered ^{15}N glycine (δiso = - 347.2 ppm). To convert to the chemical shift scale employed by protein NMR (NH_3 (l), -50 °C) it is necessary to add 379.5 ppm to the given shift.

Electrochemical measurements

The catalyst inks were prepared by mixing 20 mg of pyrolyzed PANI, or 20 mg of the 1 wt% of trapped Pt-SCC sample, or 4 mg of the 5 wt%, or 2 mg of 10 wt% trapped Pt-SCC sample or 1 mg of the 20 wt% commercial Pt/C with 5 wt% of Nafion (Sigma-Aldrich) in 4 mL of ethanol. The mixture was then ultrasonicated for 15 minutes and stirred at a rate of 500 revolutions per minute for at least 2 hours to make the catalyst suspensions.

20 µL of the suspension were dropped and coated on a glassy carbon electrode with an area of 1 cm^2. The resulted theoretical Pt loading for all Pt samples is 1 µg cm^{-2}. The catalytic activity of synthesized samples was evaluated using a three-electrode cell with a 0.05 mm thick glassy carbon (GC) plate (Sigma-Aldrich) to support the samples. A Pt metal mesh was used as counter electrode, separated from main electrolyte chamber in a fritted glass tube. A $Hg/HgSO_4$ (in saturated K_2SO_4) in a Luggin capillary was used as reference electrode and placed around 5 mm away from the working electrode. 0.5 M H_2-saturated H_2SO_4 (Suprapur) is used as the electrolyte. The reference electrode were calibrated by measuring the open-circuit potential of the Pt-mesh with continuous H_2 purging in the same electrolyte. The GC plate was polished mechanically using a 0.05 µm alumina solution in water (Buehler), followed by ultrasonication in ultra-pure water. All the electrochemical tests were recorded with a Biologic SP240 potentiostat at room temperature.

The polarization curves in the HER regime were obtained using cyclic voltammetry (CV) in the potential range between 0.1 V and -0.5 V vs. RHE at a scan rate of 10 mV s^{-1} and the scans from 0 V towards -0.5 V were plotted. The impedance of the working electrode was recorded using potentiostatic electrochemical impedance spectroscopy (PEIS) at 100 kHz and at open circuit before HER tests. A resistance of between 1 to 4 Ω was obtained for all measurements. We applied a post-measurement iR-correction to the HER polarization curves using the impedance values obtained to reveal the intrinsic activity of the Pt-SSC catalysts.

Calculation of the electrochemically active surface area

The electrochemically active surface area (ECSA) for all catalysts was estimated with the electrochemical capacitance method with equation 1 by performing CVs in the non-faradaic region.[2]

$$\mathrm{ECSA} = \frac{C_{\mathrm{dl}}}{C_{\mathrm{s}}} = \frac{i_{\mathrm{c}}}{v \cdot C_{\mathrm{s}}}, \tag{1}$$

where C_{dl} is the electrochemical capacitance, C_{dl} is the specific capacitance for a smooth

planar surface, i_c the double layer current and v the scan rate of the CVs. The CVs were carried out between 0.41 and 0.56 V vs. RHE in 0.5 M argon-purged H_2SO_4 at scan rates of 10, 20, 40, 80, 160 and 320 mV s^{-1}. The C_s value of 50 µF cm^{-2} is used for this study.[3]

Ex-situ and operando XAS

All X-ray absorption fine structure (XAFS) measurements at the Pt-L_3 edge were carried out at the B18 Core EXAFS beamline of the Diamond Light Source. For the ex-situ XAS, the Pt-PANI trapped Pt SSC, and Pt reference samples (300 mg) were finely ground, mixed homogeneously with five parts of cellulose, and pressed into 13 mm diameter pellets. Commercial dichloro[bis(pyridine)]platinum(II)($Pt(Py)_2Cl_2$, Sigma-Aldrich, 97%), $PtCl_2$ (Sigma-Aldrich, 99.995%) and PtTPP (Platinum(II) meso-tetraphenylporphine, Luminescence Technology Corp., >98%) were used as references. All spectra were recorded in transmission mode at room temperature.

The operando measurements were carried out using the Cr-coated branch of collimating and focusing mirrors, and a Si(111) double-crystal monochromator. The size of the beam at the sample position was ca. 1 mm × 1 mm. Samples were measured both in static electrolyte and operando conditions. The data were collected in fluorescence mode, by means of a 36-element solid-state germanium detector; the ion chamber at the front of the sample was used for measurement of incoming photons (I_0 filled with a mixture of 80 mbar of Ar and 1020 mbar of He to optimise sensitivity at 15% efficiency). An electrochemical cell adapted to the B18 beamline of the UK's Synchrotron Diamond Light Source was designed for the operando study shown in Supplementary Supplementary Fig. S30. Before the operando electrochemical measurements, the Pt-L_3 edge spectra were measured on contact with the liquid electrolyte. The operando XAFS spectra of the Pt-L_3 edge (11564 eV) were obtained from 200 eV before the edge up to 750 eV after the edge (corresponding to 14 $Å^{-1}$ in k-space). The measuring time was 2 minutes per spectrum. When indicated, 6 repetitions at each potential were acquired and then merged to obtain a better signal-to-noise ratio. Data were normalised

using the Athena.[4] program with a linear pre-edge and polynomial post-edge background subtracted from the raw data. EXAFS fits were performed using ARTEMIS software.[4]

Computational details

The projector augmented-wave method implemented in the Vienna Ab Initio Simulation Package (VASP)[5–7] was used for the simulations of hydrogen adsorption free energy (ΔG_{H^*}) of trapped Pt-SSC structures. A three-layer Pt-N_3-C slab model is used with the lattice constant $a = 2.477$ Å, obtained by geometry optimisation of graphite until the external stress dropped below 0.05 GPa using the revised Perdew-Burke-Ernzerhof (RPBE) functional,[8] which is known to more accurately represent the hydrogen adsorption energetics on platinum.[9] A 6 × 6 supercell of Pt-N_3-C with 12 Å of vacuum slabs were used with Grimme's D3 dispersion[10] correction to account for the vdW interaction between the graphene layers.[11] Top two layers of the Pt-N_3-C were allowed to optimise, while the third layer was fixed during the geometry optimisations. The ionic cores were described with pseudopotentials provided in VASP, where 2s and 2p were treated as valance states for carbon and nitrogen, and 5d and 6s orbitals for platinum. A 520 eV energy cutoff with a smearing of 0.1 eV and a k-point spacing of 0.2 Å were used for all calculations. We used the conjugate-gradient algorithm in VASP for the geometry optimisation.

The hydrogen adsorption free energy (ΔG_{H^*}) was calculated using the previously reported computational hydrogen electrode method by Nørskov et al.[9] with the following formalism:

$$\Delta G_{H^*} = \Delta E_H + \Delta ZPE - T\Delta S_H \tag{2}$$

where ΔE_H is the enthalpy change when one hydrogen atom is adsorbed on the N-doped graphene surface. ΔZPE is the zero-point energy difference for the hydrogen adsorption from previously reported values.[9,12,13] ΔS_H accounts for the entropy contribution of the hydrogen adsorption at 298 K, which can be approximated as $-\frac{1}{2}S^0_{H_2}$ suggested by Nørskov

et al., where $S^0_{H_2}$ is the entropy of hydrogen gas at standard conditions.[9,12] For the hydrogen adsoprtion on the Pt site in the Pt-N_3-C system, ΔE_H is obtained using Equation 3

$$\Delta E_H = \Delta E(H - Pt - N_3 - C) - E(Pt - N_3 - C) - \frac{1}{2}E(H_2). \quad (3)$$

$\Delta E(H - Pt - N_3 - C)$ is the DFT total energy of the hydrogen atom adsorbed Pt-N_3-C slab, E(Pt-N_3-C) is the total energy of the pristine Pt-N_3-C surface and E(H_2) is the DFT-calculated total energy of the hydrogen gas. The same formulism is used for the calculation of ΔG_{H^*} on N site and C site in N_3-C system.

The hydrogen absorption energy on Pt(111) was calculated to benchmark the ΔG_{H^*} of the trapped Pt-SSC. A three layer (2 × 2) Pt(111) slab model with 25 % of hydrogen coverage on the FCC site[9] of Pt(111) surface with d-spacing at 2.304 Å were employed with the top two layers allowed to relax. The ΔG_{H^*} on the FCC site of Pt(111) is 0.10 eV, which is in agreement of the value reported previously.[9] We choose to avoid the use of either explicit solvation or implicit solvation on the calculations as they are shown to marginally affect the hydrogen adsorption energetics (less than 0.01 eV), as a minor change of charge density at the Pt is to be induced by the adsorption of the hydrogen atom.[14,15]

ΔE_H, ZPE and entropy corrections for the Pt(111) surface and the Pt-N_3-C/N_3-C models are listed in table S1. Schematic drawings for Pt-N_3-C were produced using VESTA v.3.[16] Fig. 4 was plotted with the Matplotlib[17] package.

Table S1: Summary of hydrogen adsorption energy calculations.

Models	ΔE_H	$\Delta ZPE - T\Delta S_H$[9,12,13]
Pt site (Pt-N_3-C)	0.70	0.24
N site (N_3-C)	-1.07	0.44
C site (N_3-C)	-2.39	0.44
Pt(111)	-0.10	0.24

Additional results and discussion

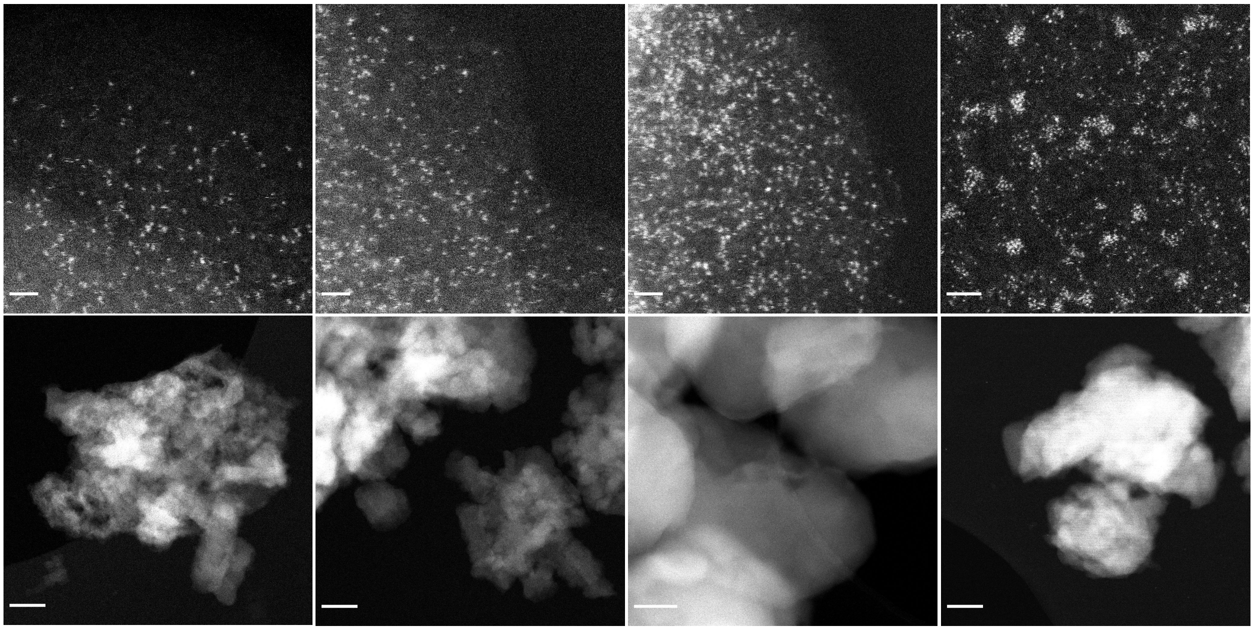

Figure S1. Atomic-resolution HAADF-STEM images show the edge positions of the 2 wt% (**a**), 5 wt% (**b**), 10 wt% (**c**), and 15 wt% (**d**) Pt-PANI samples. Low-resolution STEM images of the 2 wt% (**e**), 5 wt% (**f**), 10 wt% (**g**), and 15 wt% (**h**) Pt-PANI samples present the lack of nanoparticles over large areas of those samples. The scale bar is 2 nm in **a**-**d** and 100 nm in **c**-**f**.

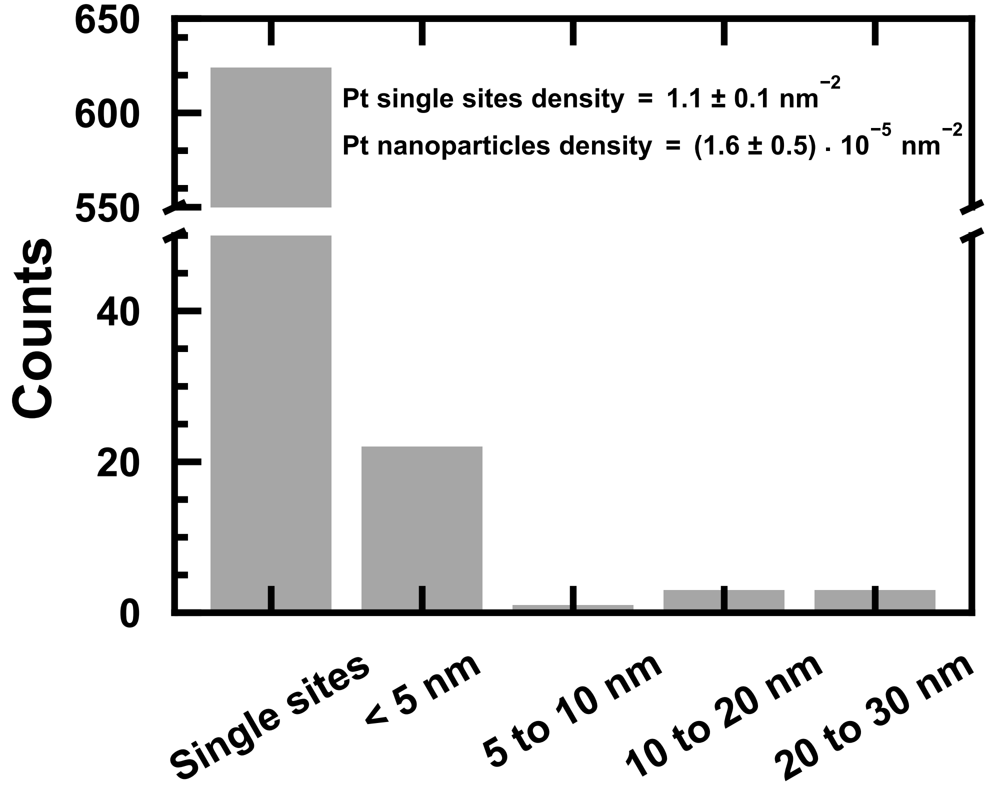

Figure S2. Size distribution of Pt single sites and Pt nanoparticles of trapped 1 wt% Pt-SSC calculated using STEM images in Figure S3. Although there are nanoparticles present in the 1 wt% Pt on polyaniline (Pt-PANI) sample after pyrolysis, we conclude that their number is statistically insignificant compared to the number of Pt single sites. We calculated the areal density of the Pt single sites and Pt nanoparticles by analysing seven STEM images of the pyrolyzed 1 wt% Pt-PANI sample. We observe that Pt single sites are five orders of magnitude denser than nanoparticles.

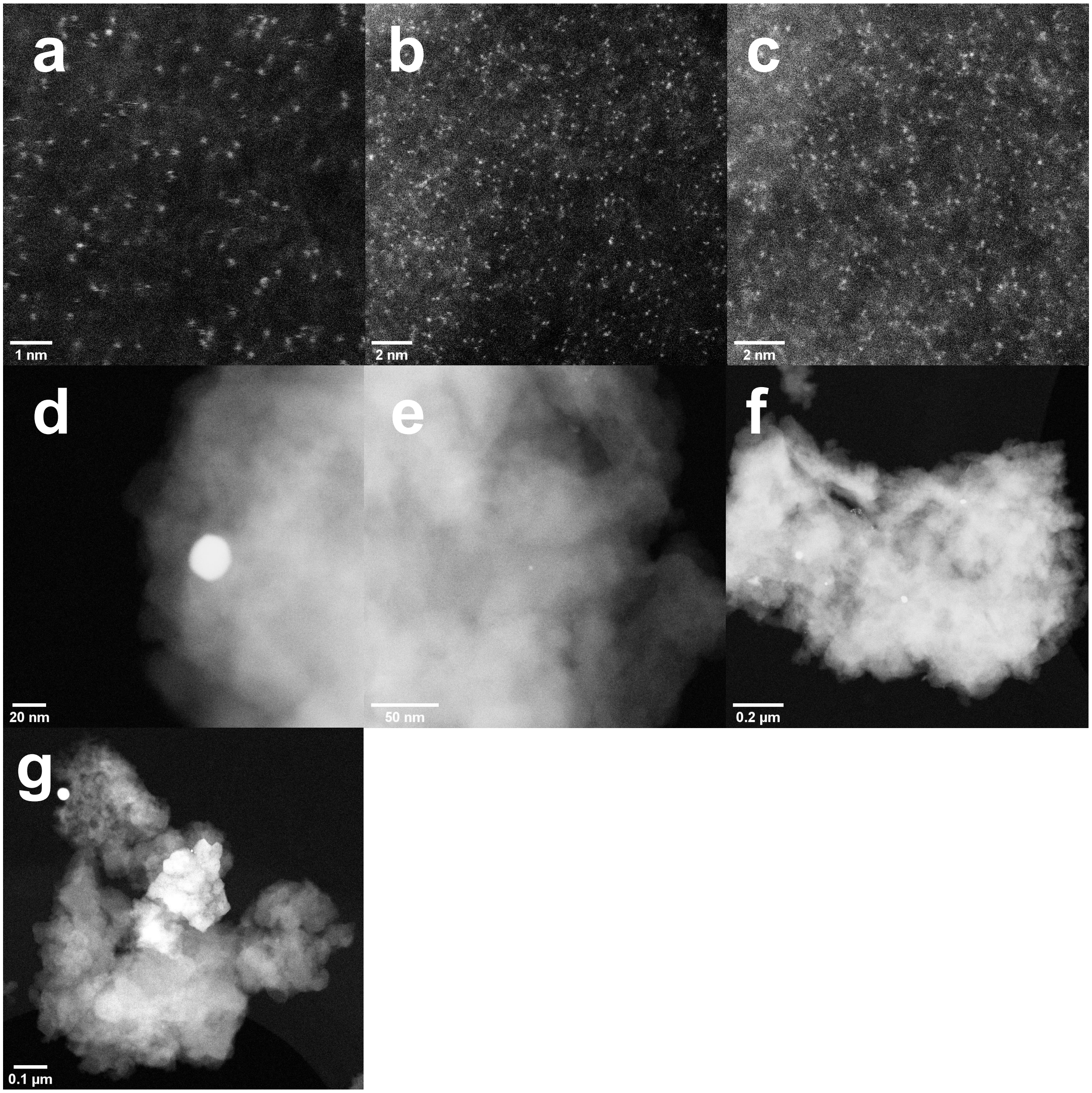

Figure S3. STEM images of pyrolyzed 1 wt% Pt-PANI used for calculation of areal density of Pt single sites and Pt nanoparticles.

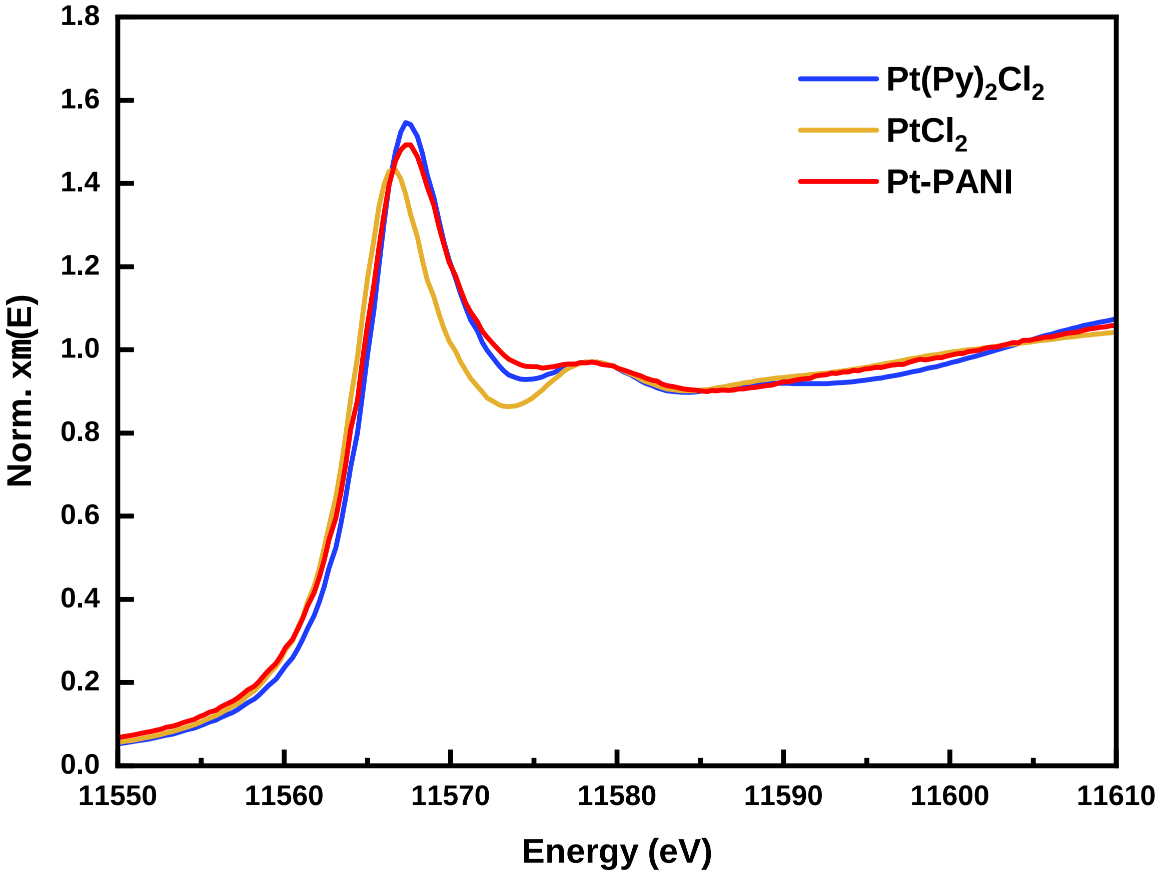

Figure S4. XANES of the Pt-PANI sample, compared with $Pt(Py)_2Cl_2$ and $PtCl_2$.

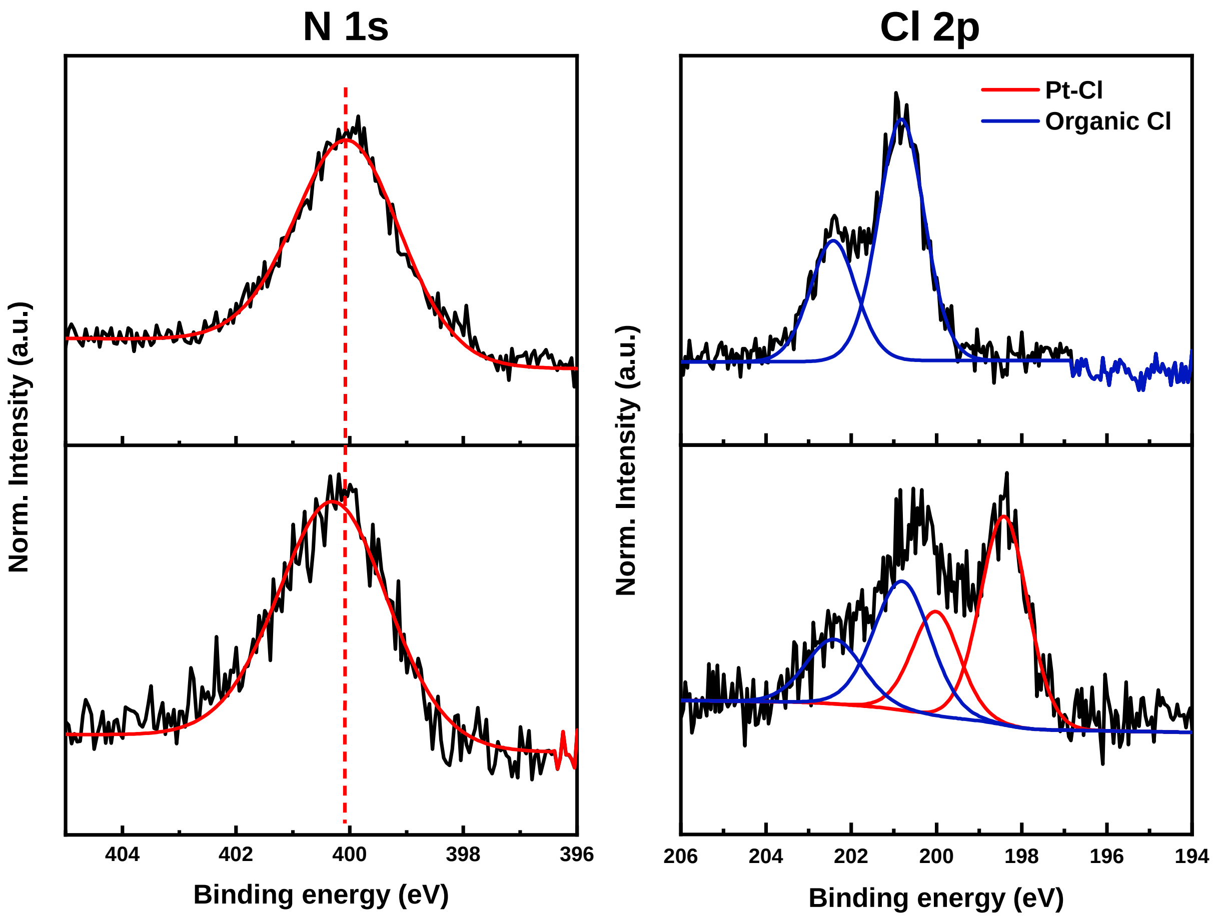

Figure S5. XPS spectra of N 1s and Cl 2p of the PANI and 10 wt% Pt-PANI samples.

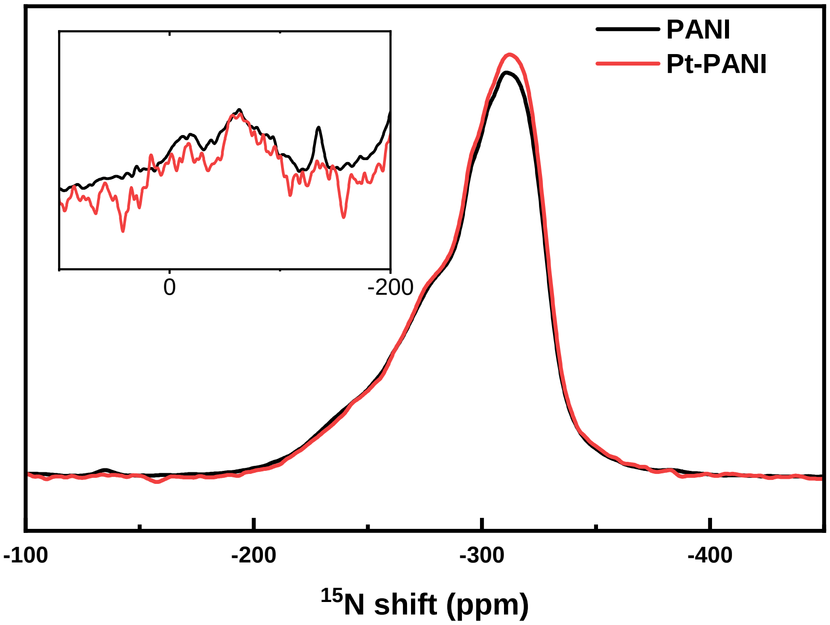

Figure S6. ^{15}N CPMAS NMR spectra of the PANI and 10 wt% Pt-PANI samples showing a slight shift of the peak after anchoring of Pt sites.

EXAFS analysis of trapped Pt-SSC

Based on a detailed analysis of the EXAFS spectra of the trapped 1 wt% Pt-SSC sample, we propose that the Pt is bonded to one carbon and three nitrogen atoms in the trapped Pt-SCC sample. We constructed five Pt-N_x-C_y models using density-functional theory (Fig. S7b) and fitted them to the experimental EXAFS data (Fig. S7a). During the fitting process, we adjusted the coordination number of Pt-N and relax all other parameters. Among the five model structures, the Pt-N_3-C model shows the lowest R factor (0.017, Table S2), indicating the best fit to the data. Therefore, we conclude the existence of a Pt-C bond in the trapped Pt-SSC.

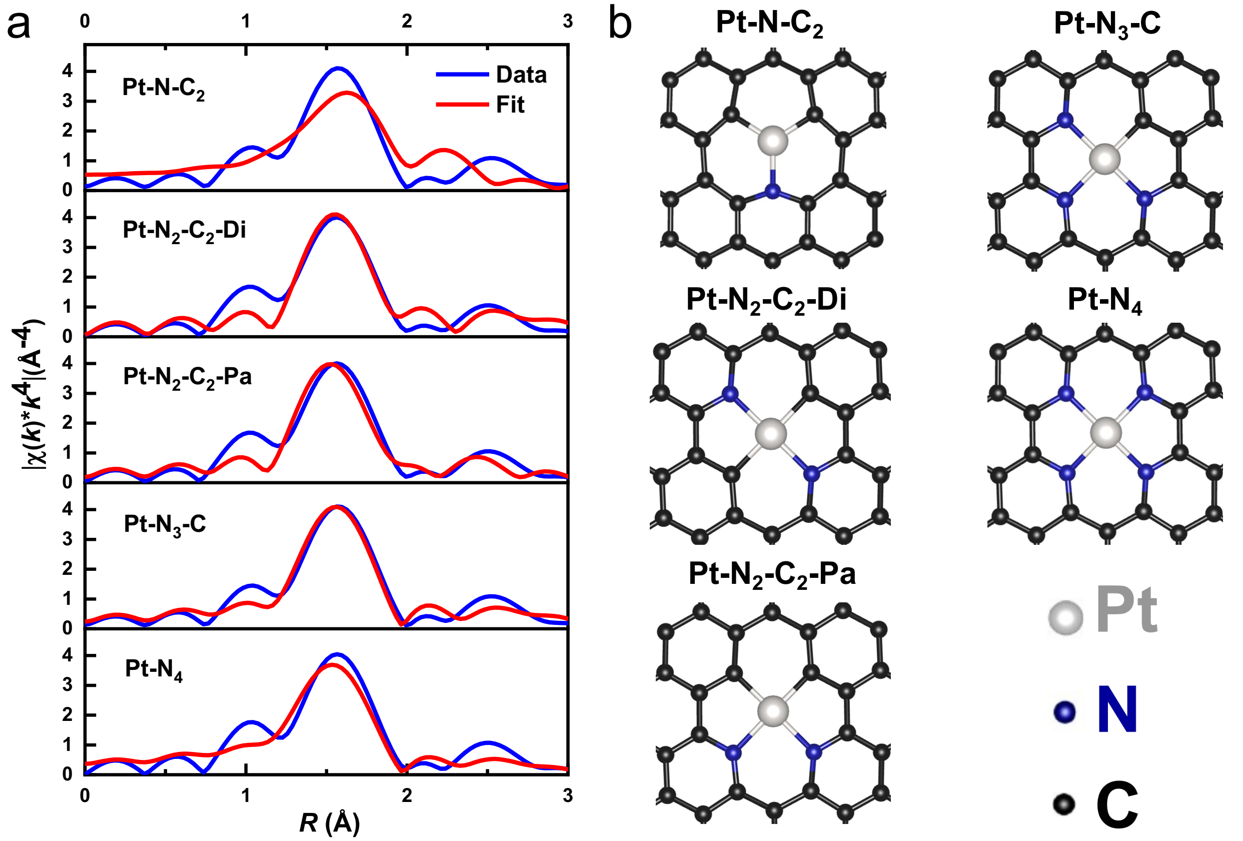

Figure S7. a) Pt L_3-edge k^3-weighted magnitude and real Fourier transform EXAFS spctra of the 1 wt% trapped Pt-SSC (blue) fitted with **b)** five different Pt-N_x-C_y structures in the range 1.1-3.0 Å in the real space (red).

Table S2: EXAFS fitting parameters of the 1 wt% trapped Pt-SSC with the data and structure models shown in Figure S7.

Models	Path	Coordination number	R-factor
Pt-N-C_2	Pt-N	1	0.086
	Pt-N-C	-314(-147.3)	
Pt-N_2-C_2-Di	Pt-N	2	0.056
	Pt-N-C	8.5(3.4)	
Pt-N_2-C_2-Pa	Pt-N	2	0.064
	Pt-N-C	17.9(8.5)	
Pt-N_3-C	Pt-N	3	0.017
	Pt-N-C	7.0(1.9)	
Pt-N_4	Pt-N	4	0.034
	Pt-N-C	5.3(3.6)	

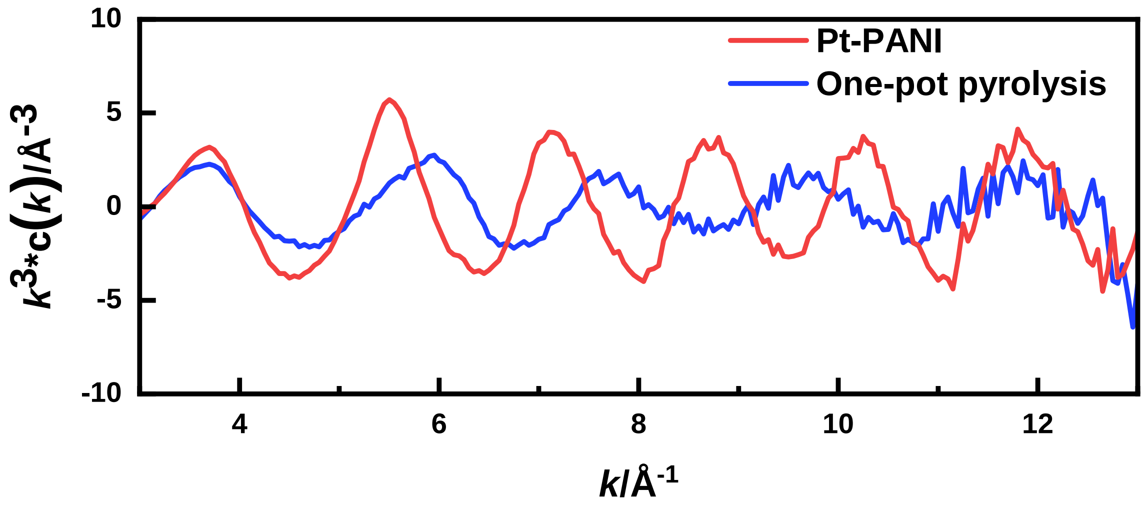

Figure S8. FT-EXAFS of 1 wt% Pt-PANI in K-space before and after pyrolysis, which clearly shows the changes in the bonding environment of Pt

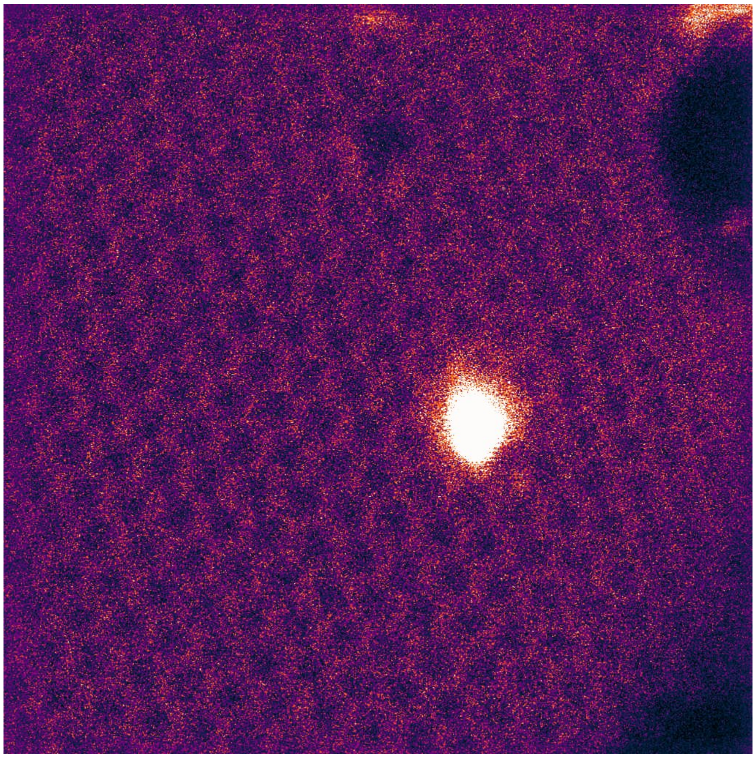

Figure S9. Atomic Z-contrast HAADF-STEM image of a Pt site supported at the surface of a defective single-layer graphene nanosheet, providing direct evidence that the use of STEM imaging to confirm the bonding between Pt sites and a graphene substrate is technically challenging due to the high contrast difference between C or N and Pt.

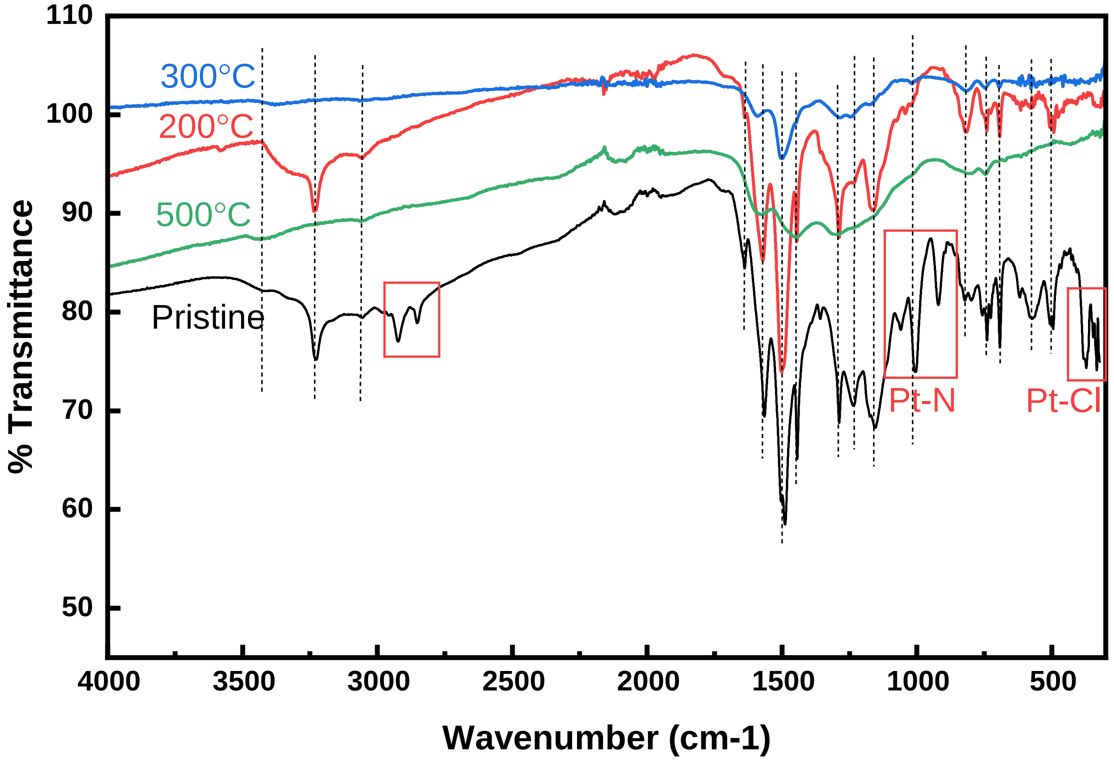

Figure S10. Ex-situ FT-IR spectra of the 10 wt% Pt-PANI sample after heating at different temperatures, showing the evolution of the PANI substrate and Pt bonding environment

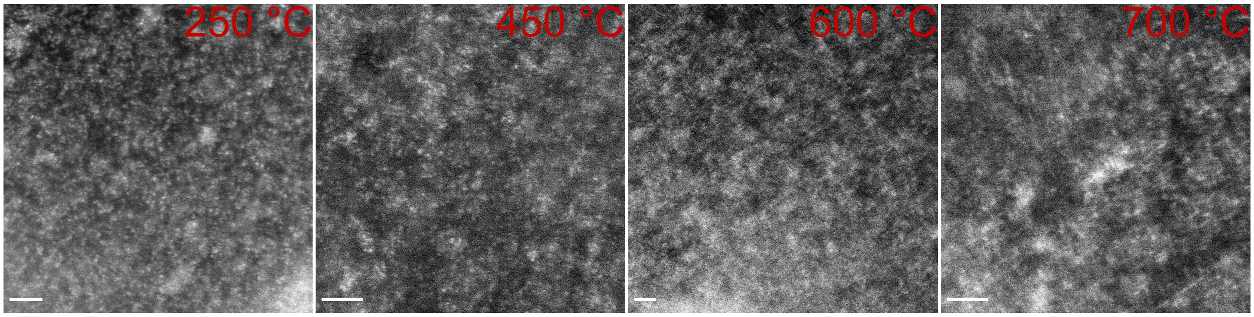

Figure S11. In-situ heated STEM images of Pt sites at the bulk areas (**a-d**) in the 10 wt% Pt-PANI sample at more heating temperatures (250 °C, 450 °C, 600 °C, and 700 °C). The scale bar is 2 nm in **a-d**.

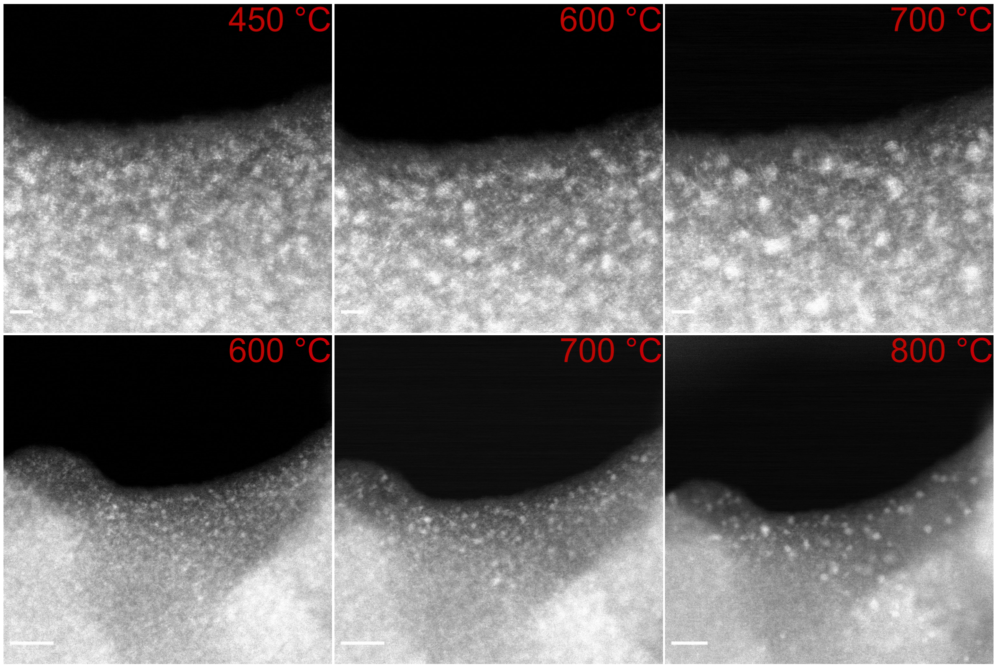

Figure S12. **a-c**, High-resolution in-situ heated STEM images of Pt sites at the edge areas in the 10 wt% Pt-PANI sample at more heating temperatures (450 °C, 600 °C, and 700 °C). The scale bar is 2 nm in **a-c**. **d-f**, Zoomed-out STEM images of Pt sites at the edge areas in the 10 wt% Pt-PANI sample at more heating temperatures (600 °C, 700 °C, and 800 °C), which show the formation of Pt nanoparticles. The scale bar is 10 nm in **d-f**.

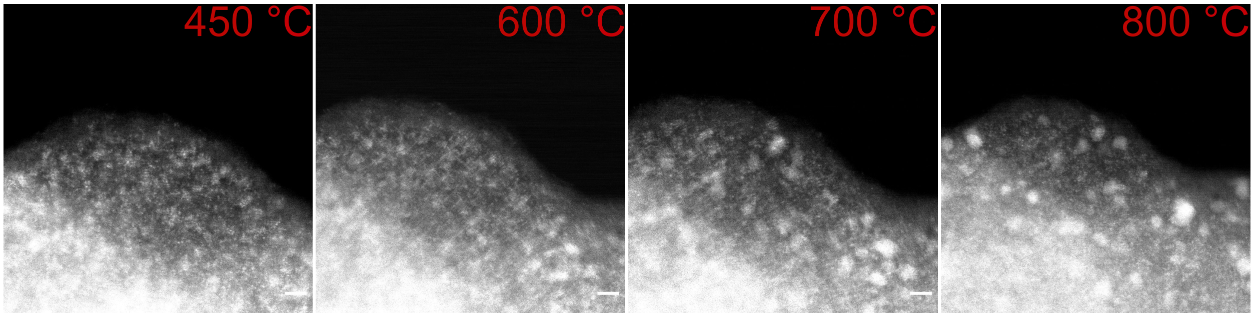

Figure S13. **a-d**, High-resolution in-situ heated STEM images of Pt sites at another edge area in the 10 wt% Pt-PANI sample at varied heating temperatures (450 °C, 600 °C, 700 °C, and 800 °C). The scale bar is 2 nm in **a-d**.

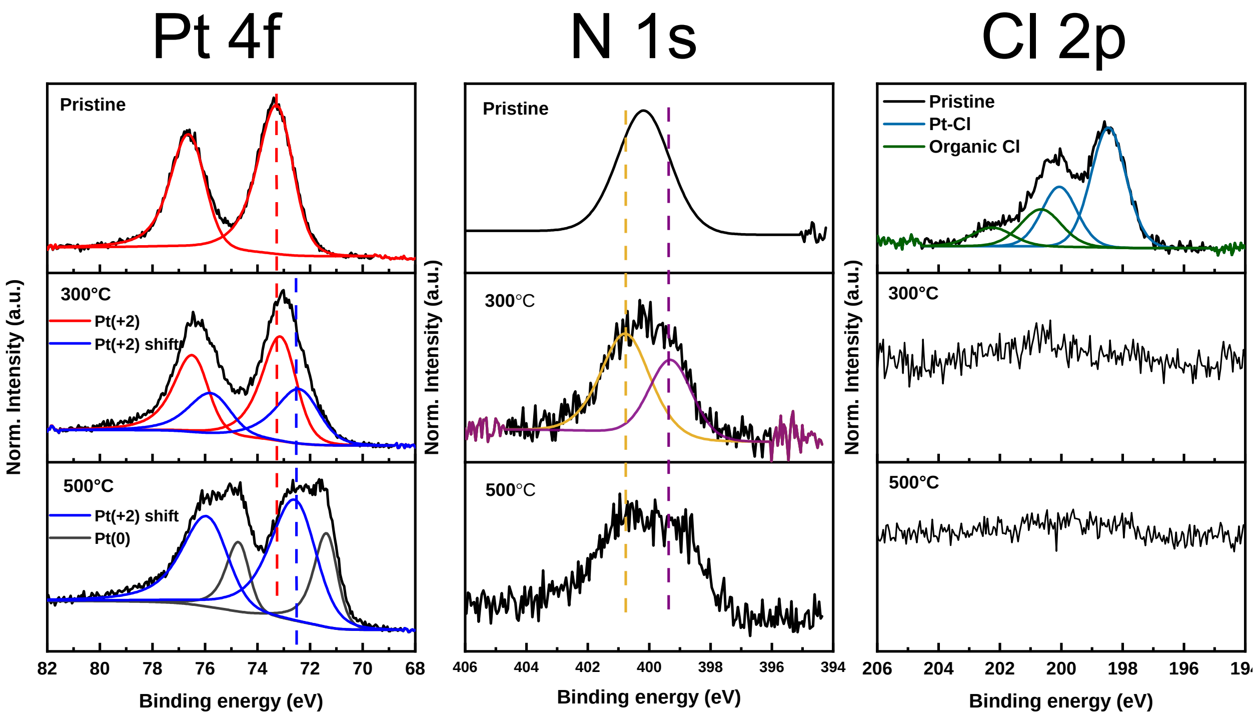

Figure S14. Ex-situ XPS spectra of the Pt 4f, N 1s and Cl 2p peaks of the 10 wt% Pt-PANI sample after heating at varied temperatures, showing the gradual reduction of Pt sites with increasing temperature

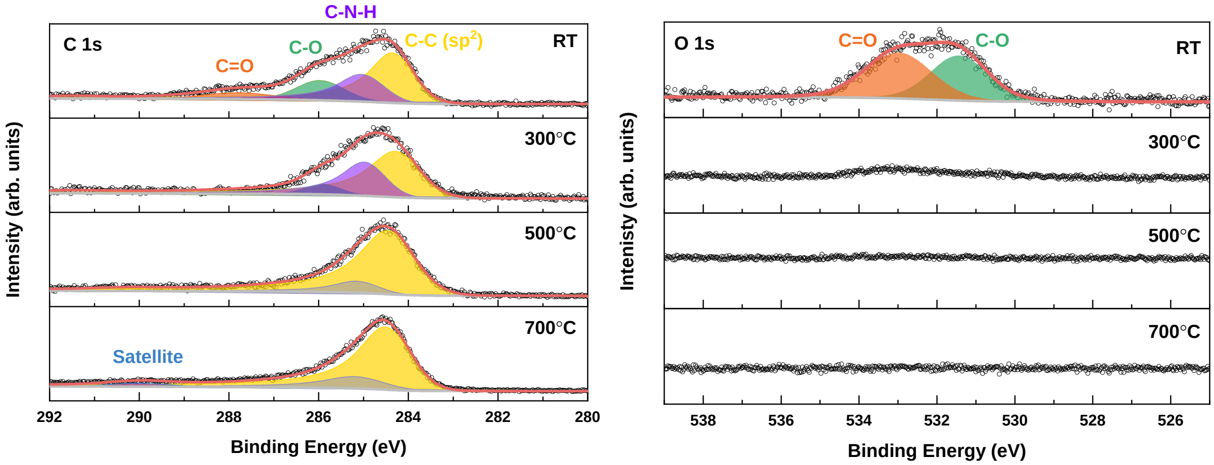

Figure S15. Temperature-dependent XPS spectra of the O 1s and C 1s peaks of the 1 wt% Pt-PANI sample at varying temperatures.

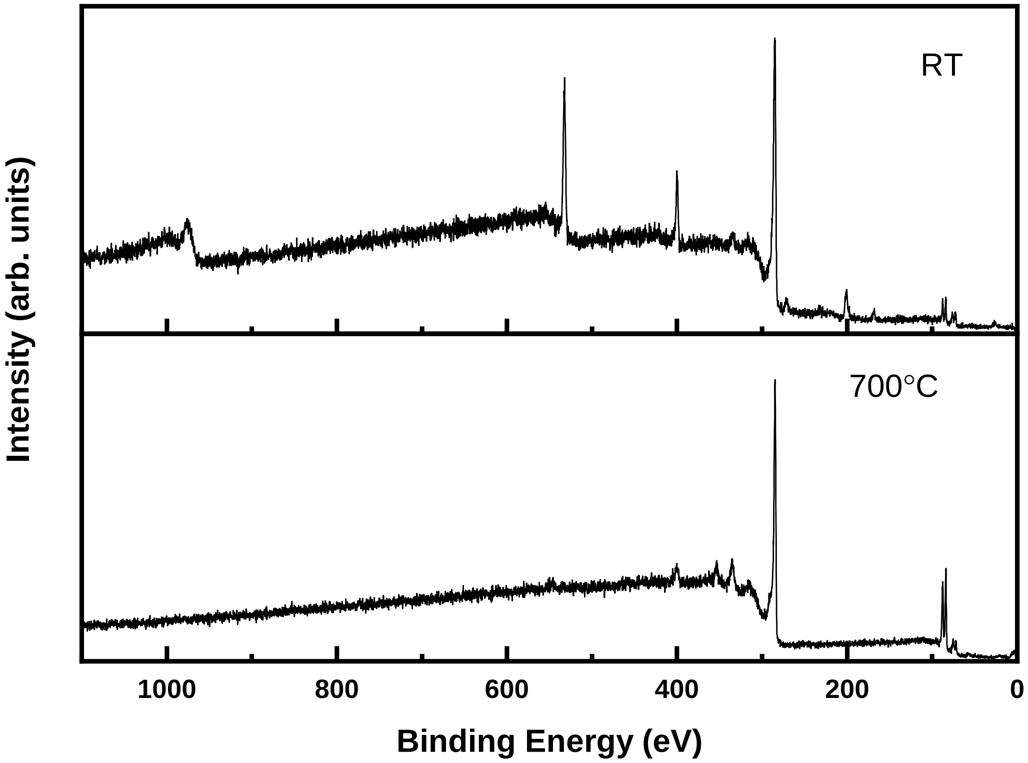

Figure S16. Temperature-dependent XPS spectra of the survey scans of the 1 wt% Pt-PANI sample at room temperature and 700 °C.

Discussions on the protocol for HER testing

HER performance for Pt-based catalysts in acidic media is prone to underestimation due to limited mass transport of the hydrogen gas produced from the catalyst surface, which can prevent the catalytic active sites maintaining contact with the electrolyte.[18] This has been reported to significantly affect the assessment of the activity of the highly HER active commercial Pt on C catalysts.[19] To allow for a fair activity comparison of our Pt SSC with commercial Pt/C, we include the following measures in our electrochemical protocol to minimise the effect resulting from mass transport of H_2.

The working electrode (WE) is placed vertically inside the cell to allow the produced H_2 to dissipate easily from the surface. An external flow of H_2 is introduced to the electrolyte solution, perturbing the solution to help accelerate the mass transport while saturating it with H_2 to establish an accurate thermodynamic onset for the HER at the WE. A low sample loading (1 µg Pt cm^{-2}) is used to limit the amount of H_2 produced, reducing the probability of the H_2 generated covering the catalyst surface. This thin coating of the catalyst also ensures thorough wetting by the electrolyte. A reference Hg/Hg_2SO_4 electrode with a Luggin capillary geometry is used and placed around 5 mm from the WE to minimise ohmic loss, allowing accurate control of the applied potential bias. The Pt-mesh counter electrode with an electrochemically active surface area (ECSA) ten times that of the active materials on the WE is used to ensure the HER current is not limited by the counter reaction. The ECSA of Pt is determined by averaging the total charge for proton adsorption and desorption, which occur between ∼0.05 and 0.4 V in a cyclic voltammetry scan in argon, and dividing by the specific charge for polycrystalline Pt (210 µC $cm^{-2}{}_{Pt}$). Supplementary Fig. S17 shows the electrochemical setup we used.

To validate our electrochemical tests, we calculated the HER turnover frequency (TOF) of the commercial 20 %wt Pt/C and compared the value with the state-of-the-art mass-transfer optimised floating electrode[20] and H_2 pump techniques.[21] The TOF is defined as the rate of H_2 produced per active site and is calculated as

$$\mathrm{TOF} = \frac{n_{H_2}}{n_{site} \cdot time} = \frac{i}{q_{HER/HOR} \cdot Q_{HUPD}} = \frac{i}{2 \cdot Q_{HUPD}} \tag{4}$$

where i is the measured HER/HOR current. Q_{HUPD} denotes the integrated charge in the HUPD experiment. $q_{HER/HOR}$ is the number of charges transferred to evolve one H_2 molecule. Using our electrochemical setup, the 20 wt% Pt/C reached a TOF value of ∼100 at -0.1 V (Supplementary Fig. S18), which is approaching the same order of magnitude as the numbers from the H_2 pump (TOF ∼1000). Specific current density curves were plotted in Supplementary Fig. S18 by normalising the current by the ECSA of the 20 wt% commercial Pt/C sample, showing that an overpotential of 50 mV is required to reach a current density of 10 mA cm^{-2}.

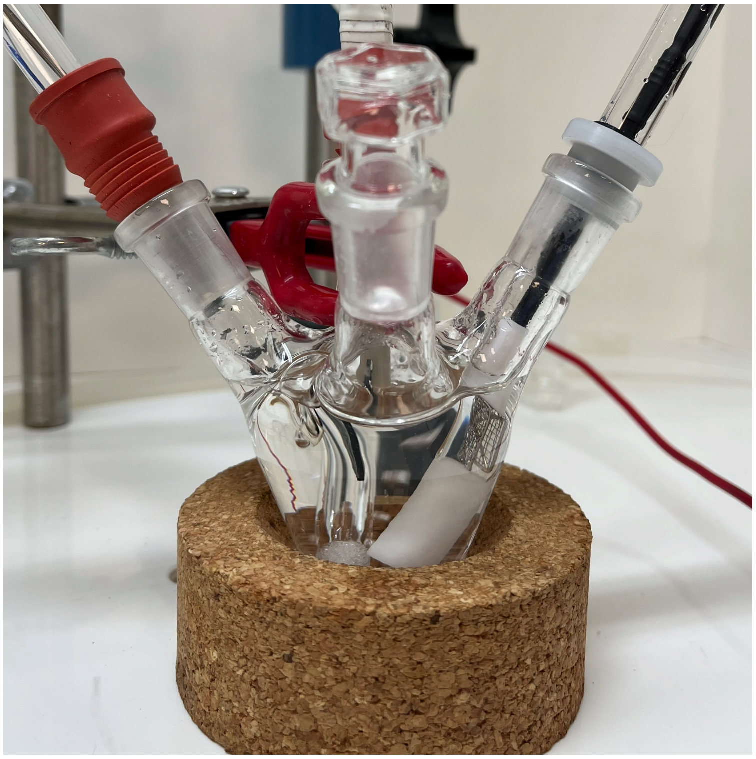

Figure S17. Photograph of the electrochemical cell used.

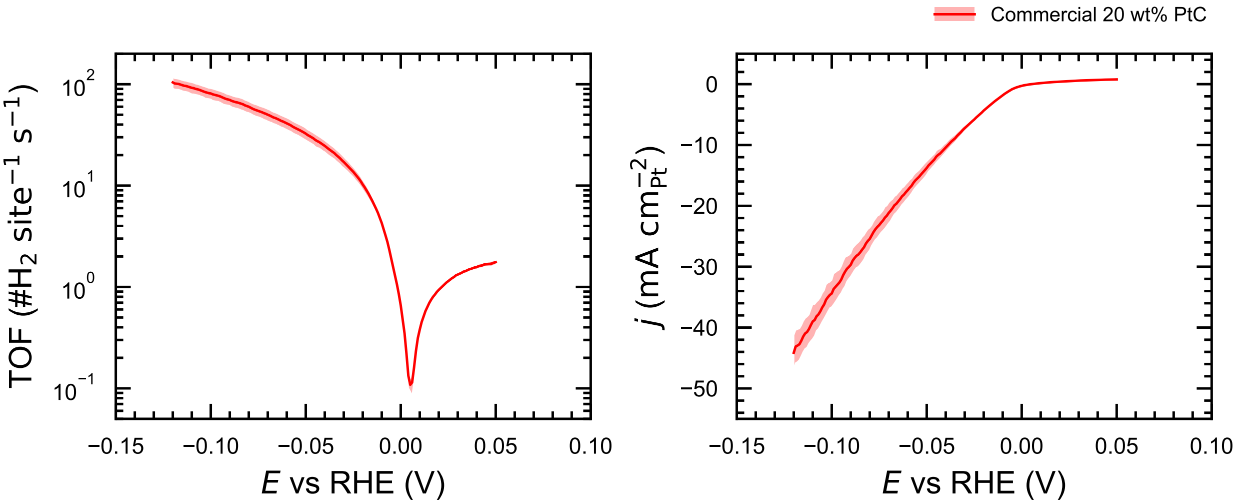

Figure S18. Turnover frequency polarization curve (**a**) and specific current density curve (**b**) of the 20 wt% commercial Pt/C measured with our electrochemical setup. The solid line is the average value from three independent measurements and the shaded area denotes the standard error.

Discussion of the HER activity of trapped Pt-SSC

We acknowledge that the Pt nanoparticles present in the trapped 1 wt% Pt-SSC sample may complicate our interpretation of the HER activity of the Pt single sites. However, we argue that the HER contribution of the trapped 1 wt% Pt-SSC shown in Figure 4 is representative of the Pt single sites, given the significant difference in both exchange current density and kinetics Tafel slope between the trapped 1 wt% Pt-SSC sample and the commercial 20 wt% Pt/C (Fig. S22).

We first normalized the HER polarization curves for each sample measured with their electrochemical active surface area (ECSA) calculated using the double layer capacitance method (Fig. S19 and Fig S20a). Then we calculated the Tafel slopes in the overpotential range of 60 to 90 mV (Fig. S21).[19] We observe an averaged Tafel slope of 160.9 ± 20.5 mV/dec for the trapped 1 wt% Pt-SSC sample compared with 124.4 ± 5.4 mV/dec for the commercial 20 wt% Pt/C from a minimum of three separate measurements (Fig. S22).

We also calculated the exchange current density for both samples by extrapolating the polarization curve from the Tafel region (between overpotential of 60 and 90 mV) to where overpotential equals zero. The current density at the intercept gives us the exchange current density of the sample (Fig. S21). We observe a j_0 for the trapped 1 wt% Pt-SSC that is four orders of magnitude lower compared with that of commercial 20 wt% Pt/C (Fig. S22).

If the Pt nanoparticles were to dominate the HER current response in the trapped 1 wt% Pt-SSC sample, we would expect to see a close agreement of the Tafel slopes and the exchange current density between the trapped 1 wt% Pt-SSC and the commercial 20 wt% Pt/C, as both of them should show the characteristic reaction thermodynamic activity and kinetics of Pt nanoparticles. As this is not the case, we think the polarization curve for the trapped 1 wt% Pt-SSC is showing the HER activity of Pt single sites.

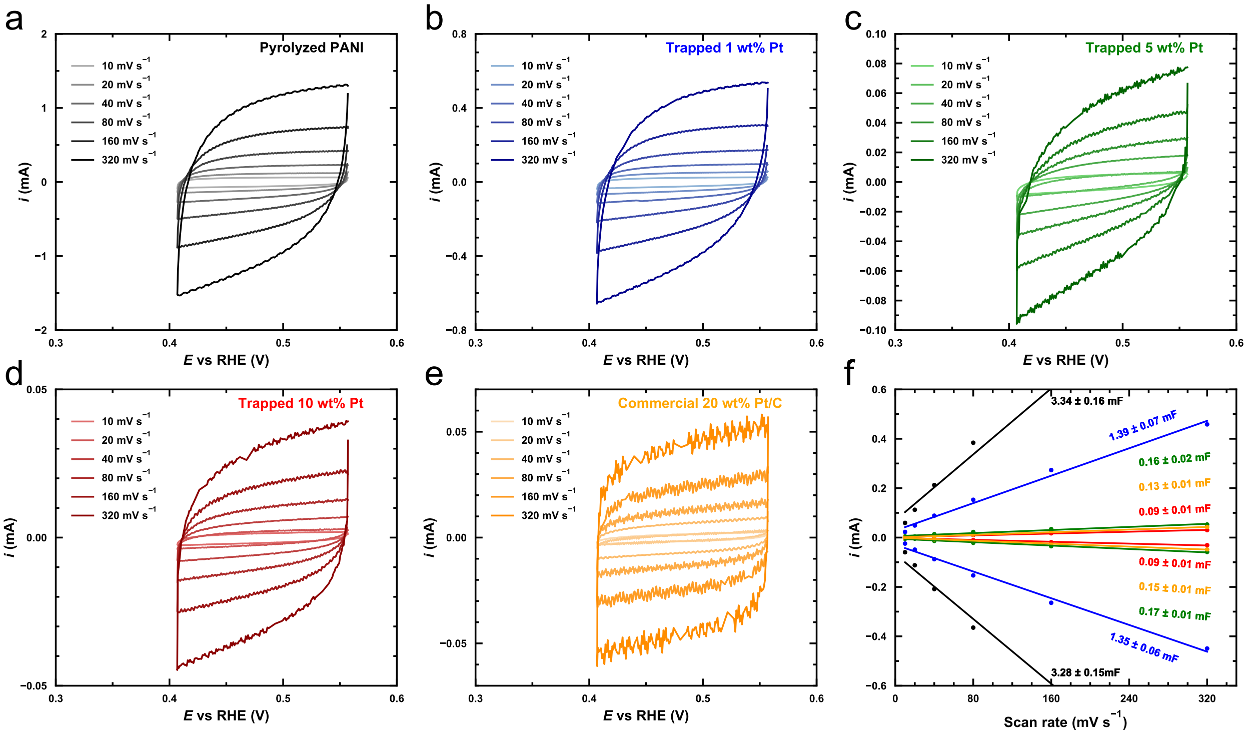

Figure S19. Electrochemical capacitance of a representative set of a) pyrolyzed PANI, b) trapped 1 wt% Pt, c) trapped 5 wt% Pt, d) trapped 10 wt% Pt and e) commercial 20 wt% Pt measurements. f) The capacitative current at 0.485 V vs. RHE plotted as a function of cyclic voltammetry scan rates with data shown in a-e.

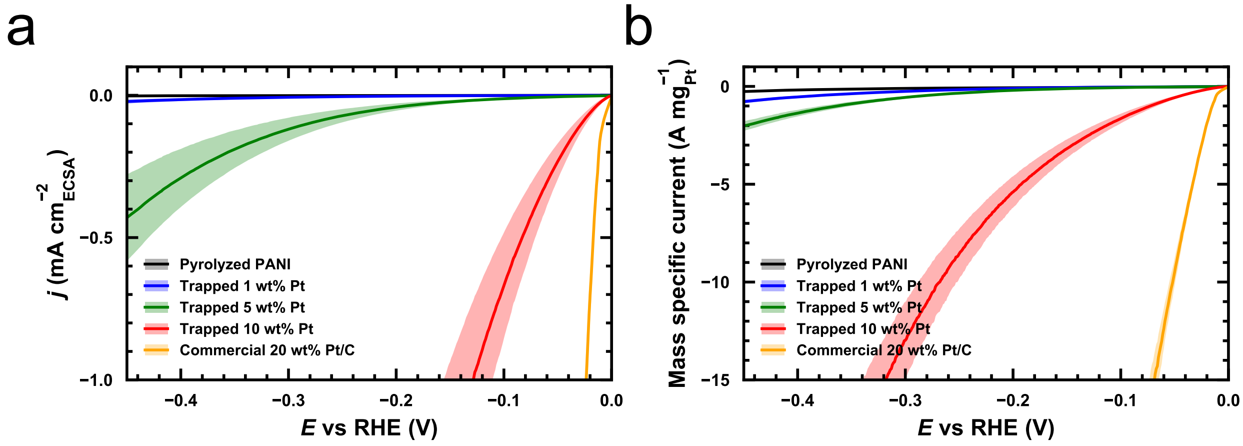

Figure S20. a) Electrochemical active surface area (ECSA) specific current density the pyrolyzed PANI, trapped 1 wt% Pt, trapped 5 wt% Pt, trapped 10 wt% Pt and commercial 20 wt% Pt and b) Pt-mass specific current of trapped 1 wt% Pt, trapped 5 wt% Pt, trapped 10 wt% Pt and commercial 20 wt% Pt. Solid curves represent the averaged current response obtained from a minimum of three separate measurements, and the shaded regions show the range of standard errors relative to the mean values.

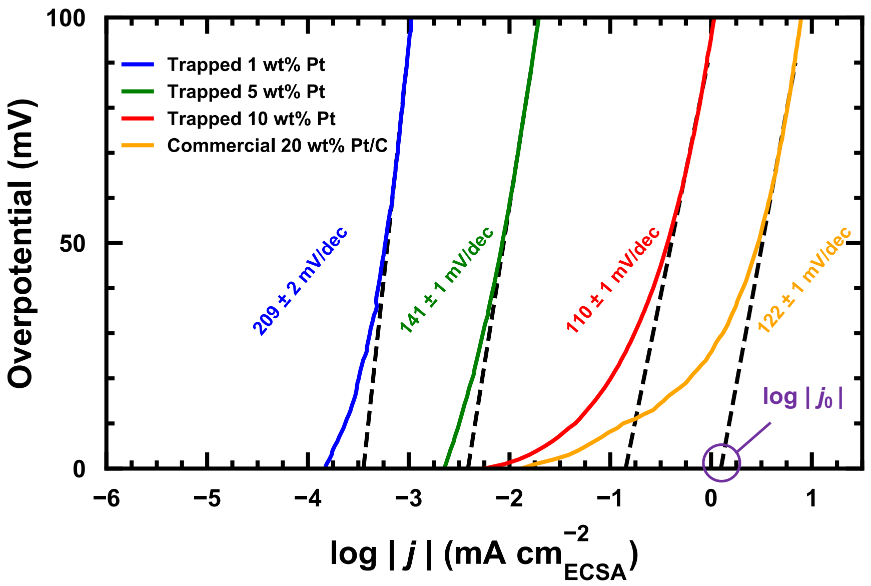

Figure S21. Tafel analysis of a representative set of HER measurement of trapped Pt-SSC samples and the commercial 20 wt% Pt/C. The dashed lines indicate linear Tafel fittings in the overpotential region between 60 and 90 mV. The extrapolation of the polarization curve from the linear Tafel region to where overpotential equals zero gives the logarithm of the exchange current density log $|j_0|$, as highlighted in purple.

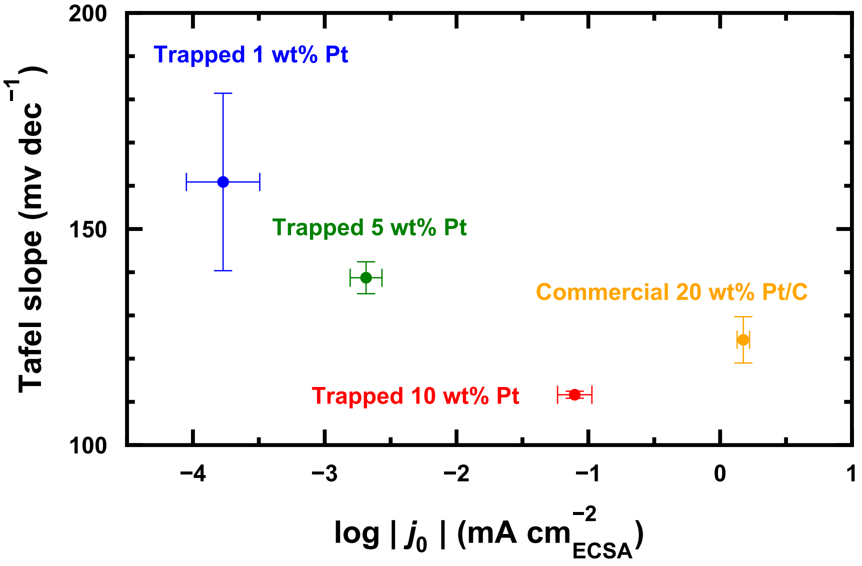

Figure S22. Tafel slopes and logarithm of the exchange current density log $|j_0|$ of trapped Pt-SSC samples and the commercial 20 wt% Pt/C. The error bars represent the standard errors obtained from a minimum of three separate measurements.

We used thermogravimetric analysis (TGA, NETZSCH STA 449 F3) to determine the mass percentage of Pt in the samples for the calculation of the mass-normalized HER current response, by burning off the carbon in the sample in oxygen and measure the weight of the remaining Pt content (Fig. S23). A heating rate of 5 °C min^{-1} from 30 °C to 900 °C was used. The weight percentages of Pt are calculated by dividing the mass at 900 °C by the mass at 150 °C, as we attribute the weight loss before 150 °C to the desorption of surface water. We obtain 2.3 wt%, 10.8 wt% and 21.7 wt% of Pt for the trapped 1 wt% Pt, trapped 10 wt% Pt and the commercial 20 wt% Pt/C, respectively. The Pt-mass specific current is calculated by normalising the current response with the total Pt mass loaded (=weight percentage of Pt times total mass of sample loaded) (Fig. S20b). We observe the same trend for the HER activity, i.e., the commercial 20 wt% Pt/C shows a superior activity to the trapped 1 wt% Pt.

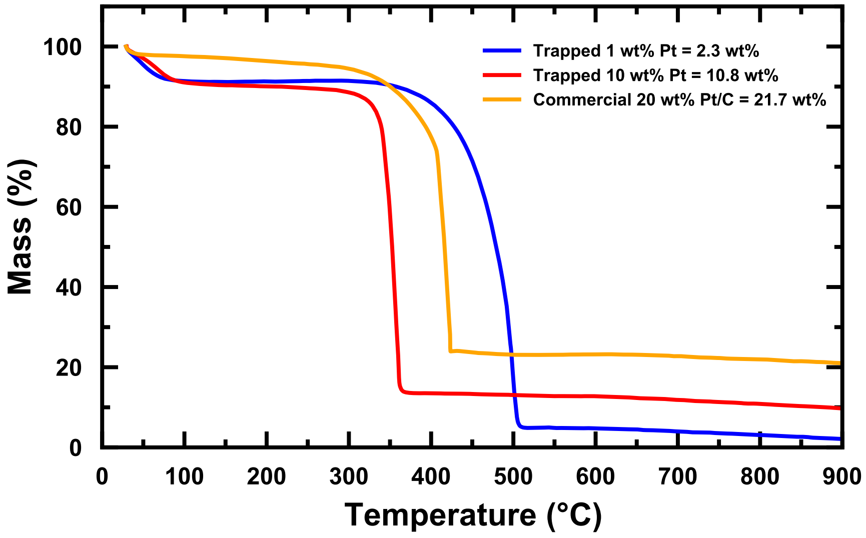

Figure S23. TGA analysis for the trapped 1 wt% Pt, trapped 10 wt% Pt and commercial 20 wt% Pt to determine the weight-percentage of Pt.

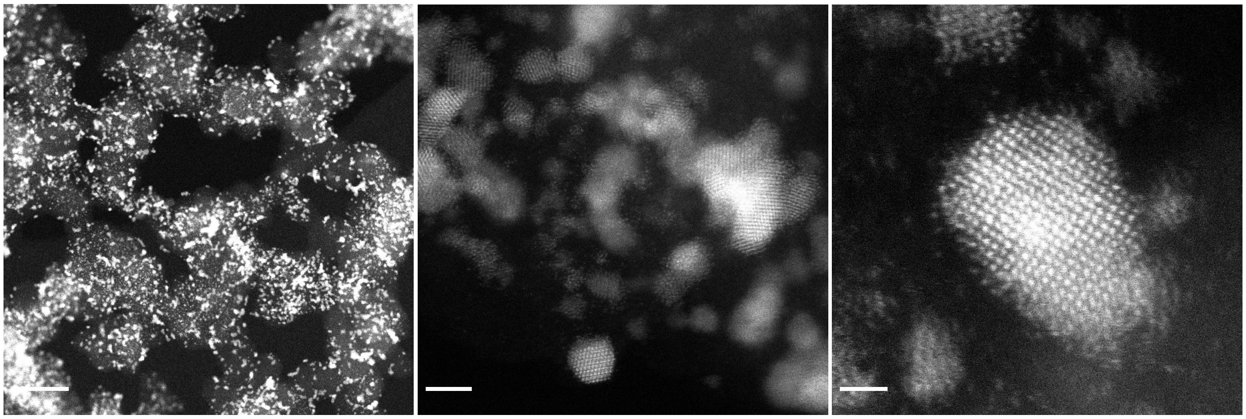

Figure S24. **a**-**c**, Low to high-resolution STEM images of the 20 wt% Pt/C showing well-dispersed Pt nanoparticles with an average size of around 3 nm on the carbon support. The scale bars are 50 nm in **a**, 3 nm in **b**, and 1 nm in **c**.

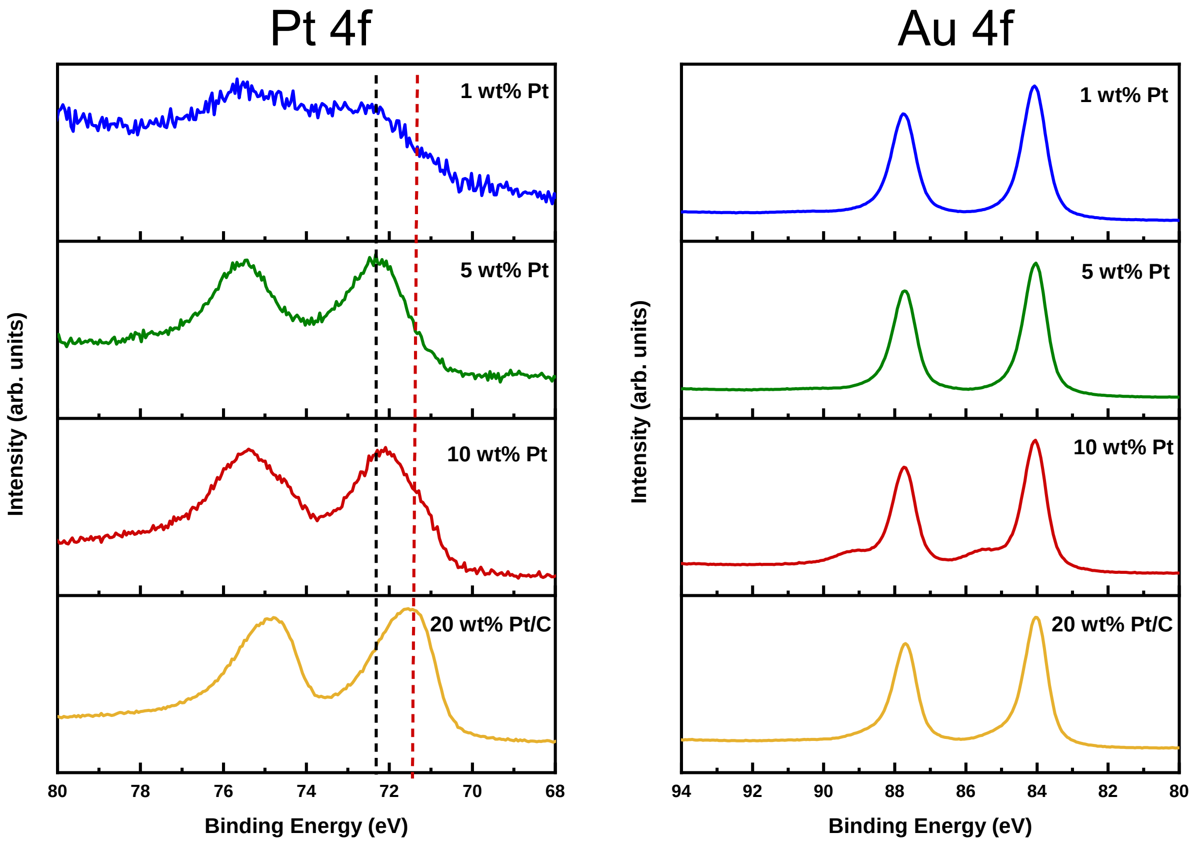

Figure S25. Ex-situ XPS data: Pt 4f and Au 4f (reference) spectra from the 1 wt% (blue), 5 wt% (green), 10 wt% (red) Pt-PANI samples after pyrolysis (the trapped Pt samples), and 20 wt% Pt/C (yellow).

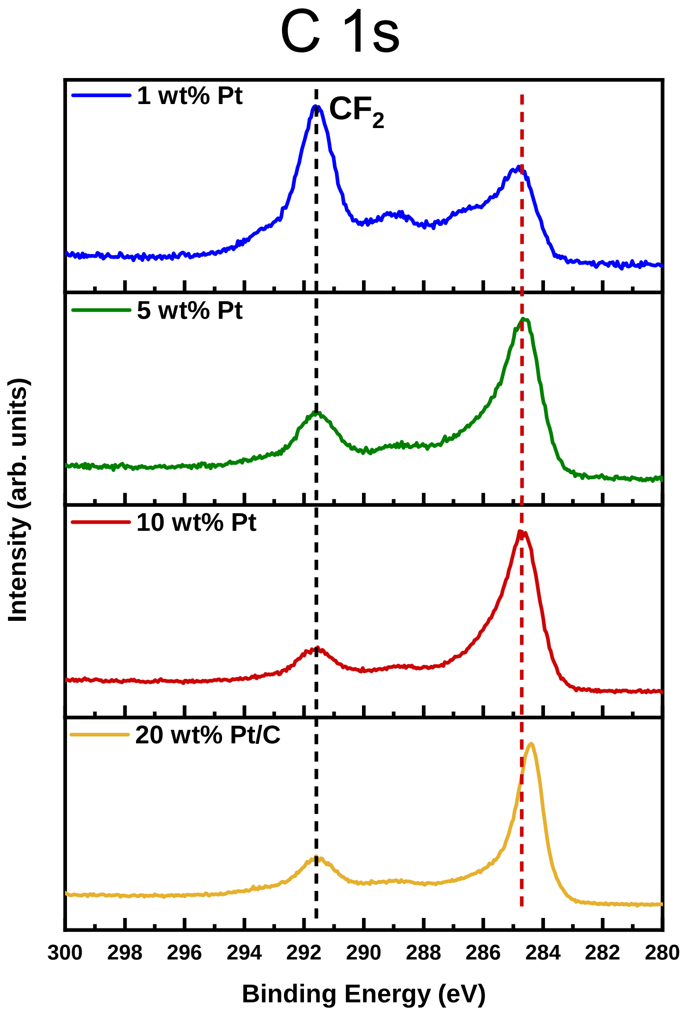

Figure S26. Ex-situ XPS spectra of C 1s from the 1 wt% (blue), 5 wt% (green), 10 wt% (red) Pt-PANI samples after pyrolysis (the trapped Pt samples) and 20 wt% Pt/C (yellow). The C 1s peaks of - CF_2 from the Nafion solution are all at 291.6 eV, which further confirms the shift in Pt 4f peaks of trapped Pt samples and 20 wt% Pt/C is reliable.

Data analysis of the operando XAS experiment

In the analysis of the operando EXAFS data, we further fix the coordination number to 3N to track the evolution of the Pt-N bond length during HER. We observed that a ∼0.01 Å shortening in the Pt-N bond length. The R-factors for fitting the operando EXAFS data at the various applied potentials using the Pt-N_3-C model remained in the range between 0.011 and 0.030, indicating a satisfactory fit with fixing the 3N coordination (Fig. S27 and Table S3).

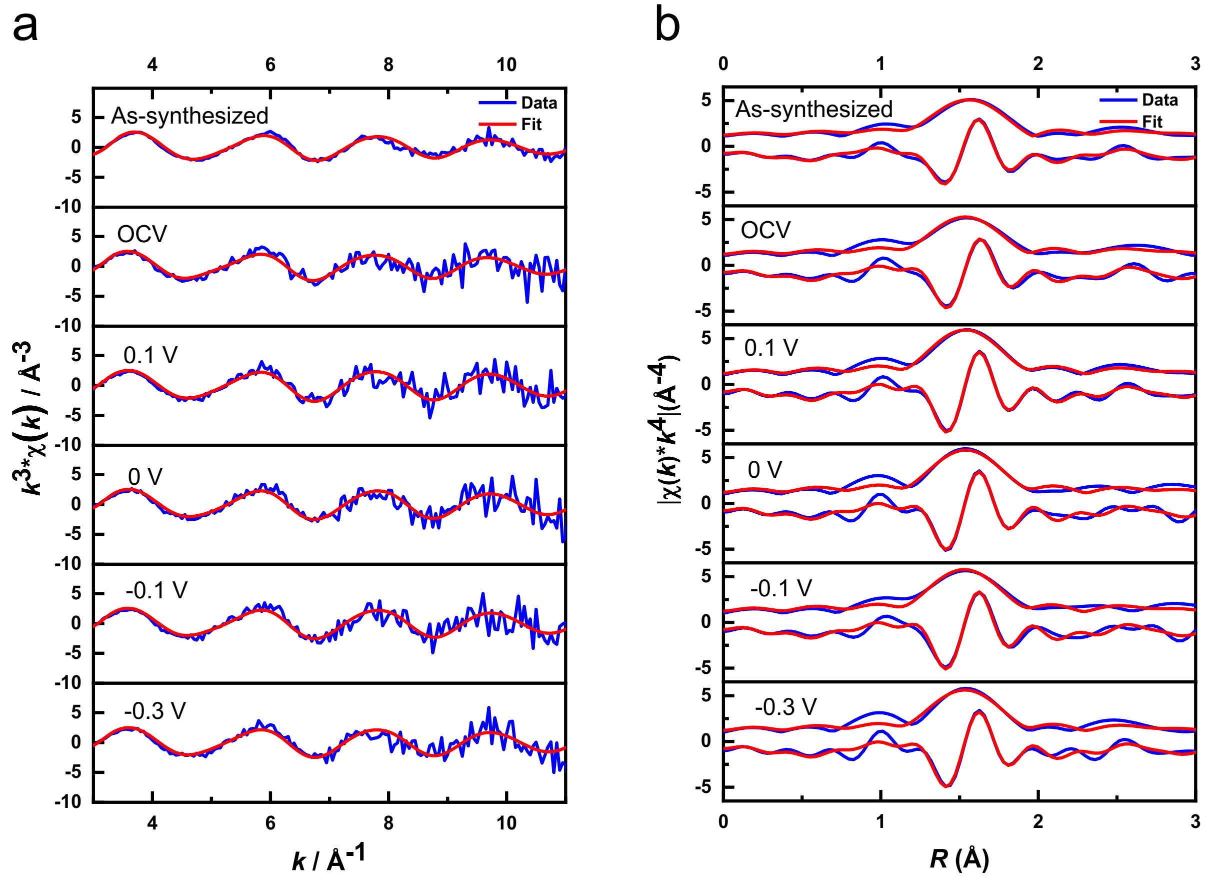

Figure S27. a) Pt L_3-edge k^3-weighted EXAFS spectra of the 1 wt% trapped Pt-SSC in the operando experiment (blue) and fitting (red) in k-space (k range: 3-11 $Å^{-1}$) using the structural model Pt-N_3-C. **b** Corresponding Pt L_3-edge k^3-weighted magnitude and real Fourier transform of a) (blue) fitted in the range of 1.1-2.4 Å in R-space (red, the as-synthesized data was fitted in the range of 1.1-3.0 Å).

Table S3: Curvefit parameters for Pt L_3-edge EXAFS data in operando experiment by DFT modelling of Pt-N_3-C and Pt foil. S_0^2 is fixed as 0.81 and the coordination number of Pt-N is fixed as 3. The fitted data range in k space and R space is: 3 – 11 Å, 1.1 – 2.4 Å for all potentials except the as-synthesized data which is 1.1 – 3.0 Å. Debye-Waller factors were constrained as δ^2(Pt-N)= δ^2(Pt-N-C) to reduce the number of variables. The numbers in brackets are uncertainties for each fitting result.

Sample	Path	CN	R(Å) enhanced	σ(10^{-3} Å^2)	R-factor
Pt foil	Pt-Pt	12	-0.010	4.4(0.2)	0.002
As-synthesized	Pt-N	3	0.019(0.010)	4.5(0.7)	0.017
	Pt-N-C	7.0(1.9)	0.151(0.044)		
OCV	Pt-N	3	0.017(0.012)	3.7(0.9)	0.026
	Pt-N-C	9.4(2.8)	0.140(0.048)		
0.10 V	Pt-N	3	0.011(0.007)	2.5(0.5)	0.011
	Pt-N-C	7.1(1.7)	0.131(0.036)		
0 V	Pt-N	3	0.012(0.010)	2.7(0.7)	0.022
	Pt-N-C	7.1(2.2)	0.156(0.048)		
-0.10 V	Pt-N	3	0.006(0.012)	2.8(0.9)	0.030
	Pt-N-C	8.3(2.8)	0.136(0.053)		
-0.30 V	Pt-N	3	0.009(0.012)	3.1(0.8)	0.028
	Pt-N-C	7.4(2.6)	0.130(0.054)		

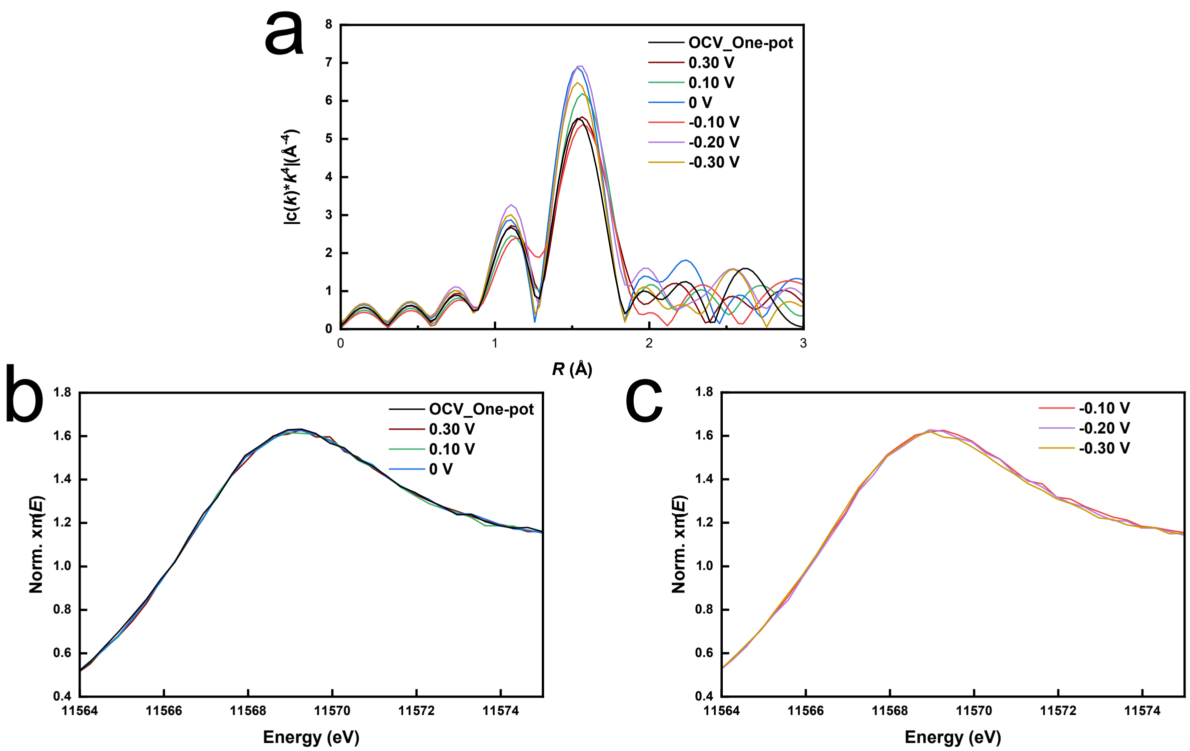

Figure S28. **a**, The FT-EXAFS spectra of Pt L_3-edge for the trapped Pt-SSC at all applied potentials. **b**,**c**, XANES spectra of Pt-L_3 edge line for the trapped Pt-SSC at applied potentials from OCV to 0 V (**b**) and -0.1 V to -0.3 V (**c**).

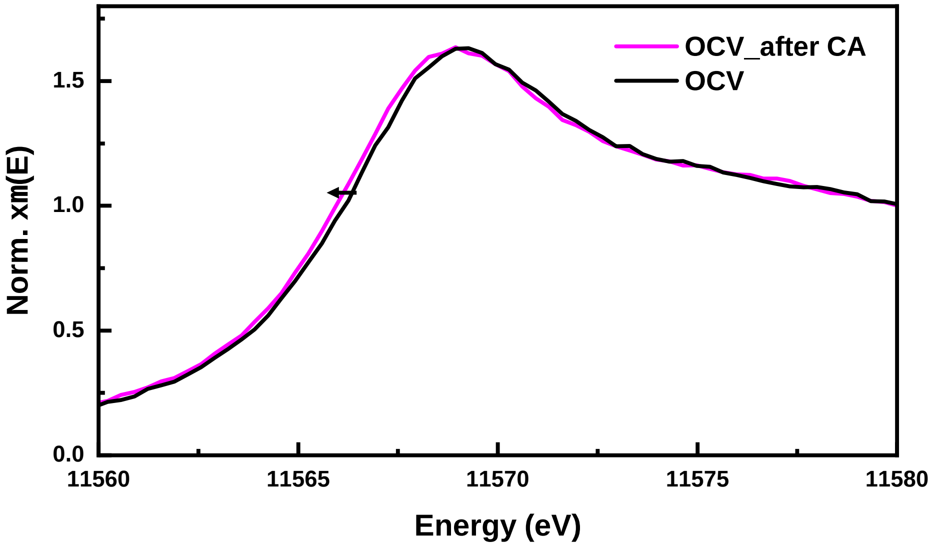

Figure S29. XANES spectra of Pt-L_3 edge line for the trapped Pt-SSC at OCV before and after 2 hrs of CA testing.

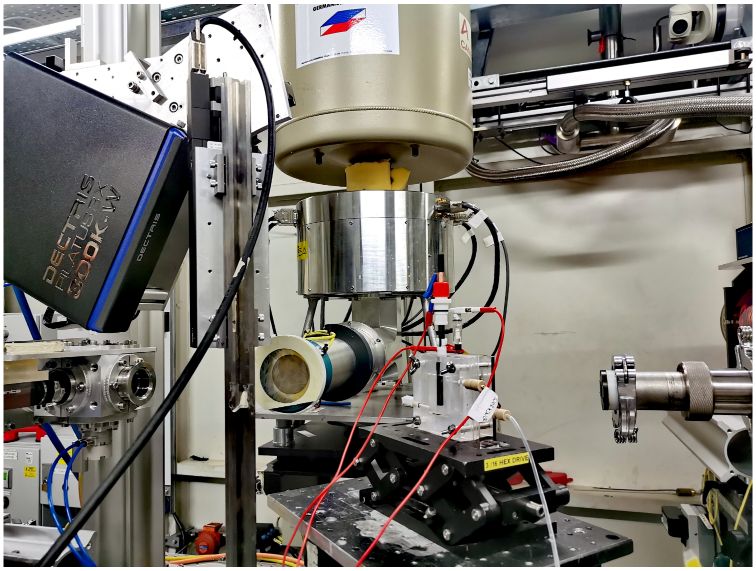

Figure S30. Photo of the operando XAS study at the Diamond beamline.

References

(1) Kaiser, S. K.; Fako, E.; Manzocchi, G.; Krumeich, F.; Hauert, R.; Clark, A. H.; Safonova, O. V.; López, N.; Pérez-Ramírez, J. Nanostructuring unlocks high performance of platinum single-atom catalysts for stable vinyl chloride production. *Nature Catalysis* **2020**, *3*, 376–385.

(2) McCrory, C. C. L.; Jung, S.; Ferrer, I. M.; Chatman, S. M.; Peters, J. C.; Jaramillo, T. F. Benchmarking Hydrogen Evolving Reaction and Oxygen Evolving Reaction Electrocatalysts for Solar Water Splitting Devices. *Journal of the American Chemical Society* **2015**, *137*, 4347–4357, PMID: 25668483.

(3) Lu, Y.; Xu, H.; Wang, J.; Kong, X. Oxygen reduction mechanism on copper in a 0.5M H_2SO_4. *Electrochimica Acta* **2009**, *54*, 3972–3978.

(4) Ravel, B.; Newville, M. ATHENA, ARTEMIS, HEPHAESTUS: data analysis for X-ray absorption spectroscopy using IFEFFIT. *Journal of synchrotron radiation* **2005**, *12*, 537–541.

(5) Kresse, G.; Joubert, D. From ultrasoft pseudopotentials to the projector augmented-wave method. *Phys. Rev. B* **1999**, *59*, 1758–1775.

(6) Kresse, G.; Hafner, J. Ab initio molecular dynamics for liquid metals. *Phys. Rev. B* **1993**, *47*, 558–561.

(7) Kresse, G.; Furthmüller, J. Efficient iterative schemes for ab initio total-energy calculations using a plane-wave basis set. *Phys. Rev. B* **1996**, *54*, 11169–11186.

(8) Hammer, B.; Hansen, L. B.; Nørskov, J. K. Improved adsorption energetics within density-functional theory using revised Perdew-Burke-Ernzerhof functionals. *Phys. Rev. B* **1999**, *59*, 7413–7421.

(9) Norskov, J. K.; Bligaard, T.; Logadottir, A.; Kitchin, J.; Chen, J. G.; Pandelov, S.; Stimming, U. Trends in the exchange current for hydrogen evolution. *Journal of The Electrochemical Society* **2005**, *152*, J23.

(10) Grimme, S.; Antony, J.; Ehrlich, S.; Krieg, H. A consistent and accurate ab initio parametrization of density functional dispersion correction (DFT-D) for the 94 elements H-Pu. *J. Chem. Phys.* **2010**, *132*, 154104.

(11) Mollenhauer, D.; Brieger, C.; Voloshina, E.; Paulus, B. Performance of Dispersion-Corrected DFT for the Weak Interaction between Aromatic Molecules and Extended Carbon-Based Systems. *The Journal of Physical Chemistry C* **2015**, *119*, 1898–1904.

(12) Atkins, P. W. *Physical Chemistry, 6th ed.*; Oxford UniversityPress: Oxford, 1998.

(13) Chan, K.; Tsai, C.; Hansen, H. A.; Nørskov, J. K. Molybdenum Sulfides and Selenides as Possible Electrocatalysts for CO_2 Reduction. *ChemCatChem* **2014**, *6*, 1899–1905.

(14) Rossmeisl, J.; Nørskov, J. K.; Taylor, C. D.; Janik, M. J.; Neurock, M. Calculated Phase Diagrams for the Electrochemical Oxidation and Reduction of Water over Pt(111). *The Journal of Physical Chemistry B* **2006**, *110*, 21833–21839, PMID: 17064147.

(15) Sakong, S.; Naderian, M.; Mathew, K.; Hennig, R. G.; Groß, A. Density functional theory study of the electrochemical interface between a Pt electrode and an aqueous electrolyte using an implicit solvent method. *The Journal of Chemical Physics* **2015**, *142*, 234107.

(16) Momma, K.; Izumi, F. VESTA 3 for three-dimensional visualization of crystal, volumetric and morphology data. *Journal of applied crystallography* **2011**, *44*, 1272–1276.

(17) Hunter, J. D. Matplotlib: A 2D graphics environment. *Computing in Science & Engineering* **2007**, *9*, 90–95.

(18) Hansen, J. N.; Prats, H.; Toudahl, K. K.; Secher, N. M.; Chan, K.; Kibsgaard, J.; Chorkendorff, I. Is There Anything Better than Pt for HER? *ACS Energy Letters* **2021**, *6*, 1175–1180.

(19) Zheng, J.; Yan, Y.; Xu, B. Correcting the Hydrogen Diffusion Limitation in Rotating Disk Electrode Measurements of Hydrogen Evolution Reaction Kinetics. *Journal of The Electrochemical Society* **2015**, *162*, F1470–F1481.

(20) Zalitis, C. M.; Kramer, D.; Sharman, J.; Wright, E.; Kucernak, A. R. Pt Nano-Particle Performance for PEFC Reactions at Low Catalyst Loading and High Reactant Mass Transport. *ECS Transactions* **2013**, *58*, 39.

(21) Durst, J.; Simon, C.; Hasché, F.; Gasteiger, H. A. Hydrogen Oxidation and Evolution Reaction Kinetics on Carbon Supported Pt, Ir, Rh, and Pd Electrocatalysts in Acidic Media. *Journal of The Electrochemical Society* **2015**, *162*, F190–F203.
